# Beyond Self-Assembly: Bioorthogonal ‘Click’ Chemistry Strategies for Robust Electrochemical Interfaces in Wearable Biosensors

**DOI:** 10.3390/bios16030181

**Published:** 2026-03-23

**Authors:** Roy Merkezoğlu, Özgür Yılmaz, Ahmet Akif Kızılkurtlu

**Affiliations:** 1 Division of Organic Chemistry, Department of Chemistry, Faculty of Engineering, Istanbul University-Cerrahpaşa, Istanbul 34320, Türkiye; 2 Marmara Research Center, The Scientific and Technological Research Council of Türkiye (TUBITAK), Gebze 41470, Türkiye; 3 Department of Pharmaceutical Chemistry, Faculty of Pharmacy, Istanbul University, Istanbul 34116, Türkiye; 4 Department of Biomedical Engineering, Faculty of Engineering and Natural Sciences, Istanbul Atlas University, Istanbul 34403, Türkiye

**Keywords:** bioorthogonal chemistry, click chemistry, wearable biosensors, electrochemical interfaces, surface functionalization, bioconjugation

## Abstract

Electrochemical biosensors integrated into wearable devices have revolutionized the technology in terms of health monitoring and diagnostic systems. However, when it comes to moving the devices from the laboratory to real-world environments, a critical problem emerges with the interface. The problem, in essence, is that biorecognition elements tend to lose their activity, delaminate, and drift when exposed to various environmental stresses. The traditional methods for the immobilization of the biorecognition elements result in receptors with random orientations, hydrolytically unstable bonds, and batch-to-batch variability, regardless of the method, including physisorption or non-selective covalent attachment, like using EDC/NHS. This review is organized around a comparative question: which limitations of classical immobilization strategies (physisorption, self-assembled monolayers used as passive anchoring platforms, and EDC/NHS coupling) can be resolved by click chemistry, which can be resolved by mechanistic features? Accordingly, CuAAC, SPAAC, IEDDA, and thiol-ene/yne photoclick reactions are discussed, not as an isolated catalog of ligations, but as complementary solutions to specific interfacial failure modes, including random bioreceptor orientation, hydrolytically vulnerable attachment, poor batch reproducibility, catalyst sensitivity, and the difficulty of functionalizing soft polymeric or textile substrates. In this framework, click chemistry is treated as a deterministic interface-engineering strategy that enables defined covalent fixation, programmable probe density, and improved mechanical and electrochemical robustness under wearable operating conditions.

## 1. Introduction

The change in modern medicine is moving away from episodic and centralized diagnostics and toward decentralized and continuous monitoring. This is being driven by the advancing technology of wearable bioelectronics and augmented by the development of bioorthogonal chemistry [1,2]. Wearable sensors are designed to either sit on the skin or be integrated into the fabric of clothes. They are designed for the noninvasive monitoring of physiological biomarkers in biofluids such as sweat, saliva, and interstitial fluids [3]. Despite the significant advances in the development of flexible substrates and signal-processing electronics for wearable sensors, their commercialization and practical applications are still being impeded by the so-called “interface bottleneck.” The interface bottleneck is the inherent instability and unreproducible nature of the biotic–abiotic interface between the biological recognition element and the inorganic electrode transducer [4]. Unlike the stable and controlled environments of bench-top analytical instruments, wearable sensors are required to operate in dynamic and fluctuating physiological environments. They are required to withstand mechanical stress and temperature changes. They are also required to operate in complex fouling environments. All these requirements demand interfacial robustness, which is not provided by the conventional methods of immobilization [5]. The conventional approach to the development of electrochemical biosensors is based on the choice of the method of immobilization based on simplicity rather than on the structural integrity and orientation of the molecules. The conventional methods of immobilization are mostly based on either physical adsorption or random covalent cross-linking [6]. The thermodynamic and kinetic limitations of the mentioned conventional methods are the main hindrances in the development of high-performance sensors.

Physical adsorption (physisorption) is the easiest method for immobilizing the bioreceptors on the surfaces of electrodes, where effectiveness and stability heavily depend on the surface properties of the electrode. This method uses weak, noncovalent forces such as electrostatic attraction, hydrogen bonding, and van der Waals forces to bind the bioreceptor to the electrode [7]. Initially, the method helps preserve the native structure of the bioreceptor. However, the binding energy is low [8]. This causes the bioreceptor to leach when the sensor is subjected to the fluid, whether flowing or steady [9], which is not desirable for continuous monitoring. In addition, when the bioreceptors are immobilized onto the sensor, their conformation changes, leading to an increase in the amount of contact between the bioreceptor and the sensor. The interaction results in the bioreceptor denaturing over time. To improve the stability of the interfaces, the application of covalent coupling by utilizing ethyl-3-(3-dimethylaminopropyl) carbodiimide (EDC) and N-hydroxysuccinimide (NHS) has emerged, and is now considered the standard approach [10]. The background of this method is the principle of reacting primary amines, specifically as the lysine group found in the protein, in order to form amide linkages with carboxylated electrodes [11]. However, the implementation of the technique brings some considerable disadvantages. One of the main problems is hydrolytic instability; this takes place when the O-acylisourea intermediate of the reaction is hydrolyzed in an aqueous solution, resulting in the low efficiency and poor reproducibility of the interfaces. For example, in a study conducted with graphene-based interfaces, it was found that the utilization of the EDC and NHS reagents is not suitable and often fails to effectively functionalize the pristine carbon material without introducing defects in the structure. It must be noted that it negatively impacts the electrical conductivity of the material [12]. Another problem is the random orientation of the molecules on the surface. The protein contains several lysine groups on the surface [13]. The application of the EDC and NHS reagents is not specific to particular sites on the protein. The result is a random population of molecules in which the active sites are often blocked [14]. The application of click chemistry is designed to circumvent the problems of randomness and hydrolytic instability. To overcome the stochastic nature and instability of classical interfaces, the field is pivoting toward bioorthogonal “click” chemistry [15]. Click chemistry is a term coined by Sharpless and his group for a set of chemical reactions that are modular in nature, used in broad-scope, high-yielding, and stereospecific applications [16]. For the application of click chemistry in the development of biosensors, the term “bioorthogonal” is used to describe the ability of the chemical reaction to take place in a complex biological system without affecting the native biochemistry or the myriad functional groups present in the system [17].

The adoption of bioorthogonal strategies, such as Cu(I)-catalyzed azide–alkyne cycloaddition (CuAAC), strain-promoted azide–alkyne cycloaddition (SPAAC), and Inverse Electron Demand Diels–Alder (IEDDA) reactions, offers distinct advantages [18]. First, by incorporating bioorthogonal handles (e.g., azides or alkynes) at specific amino acid residues or glycan chains, bioreceptors can be immobilized in a predetermined, uniform orientation [19]. The directed assembly procedures provide for active sites to interact with the analyte solution, which results in enhanced sensitivity and better limit of detection (LOD) compared to casual methods [20]. Secondly, the resulting linkages, such as the 1,2,3-triazole ring formed in CuAAC, are chemically inert and extremely stable against hydrolysis, oxidation, and enzymatic degradation. It is not present in the labile amide or ester bonds formed by traditional cross-linkers [21]. Therefore, covalent stability is critical for preventing bioreceptor detachment under the mechanical stress of wear-based implementations. Finally, the click chemistries like CuAAC create conjugated triazole linkers that can facilitate electron transfer, acting as molecular wires rather than insulating barriers [22]. This is further enhanced using clickable graphene nanoribbons (GNRs), which provide a robust, conductive scaffold for high-fidelity electrochemical sensing [23].

**Figure 1:**
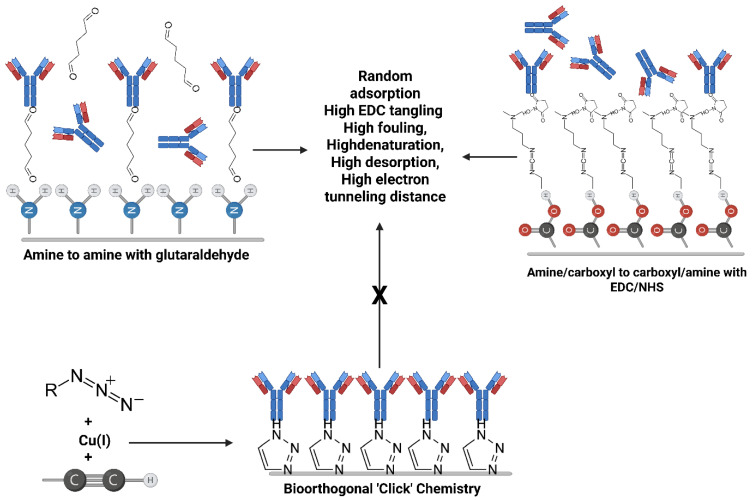
The common problems in most known linking chemistry strategies and the advantages of click-chemistry-based immobilization techniques.

In this review, we deeply examined the field of bioorthogonal chemistry in the context of constructing robust electrochemical interfaces for wearable biosensor applications. Furthermore, we discussed the rates of reaction mechanisms and identified the best click chemistry approaches to use with different wearable sensor materials and biological receptors; a comparison of CuAAC, SPAAC, and IEDDA was performed. In addition, we evaluated the molecular engineering approaches in interface design in terms of designing conductive linkers like graphene nanoribbons and the incorporation of antifouling spacer groups like zwitterionic peptides to demonstrate signal quality in complex biofluid [24]. Moreover, it was aimed to demonstrate how such detailed chemistry plays a critical role in determining key electrochemical interface performance parameters such as sensitivity, long-term durability, and mechanical robustness [25]. Finally, the emerging challenges related to steric effects in dense bioorthogonal monolayers and toxicity of catalysts in click chemistry approaches were explained. The vision for the latest wearable diagnostics technologies towards translation to the clinic was also presented. Figure 1 shows a schematic comparison of bioorthogonal chemistry with traditional linker chemistry.

Throughout this review, the phrase “beyond self-assembly” is used in a precise interfacial sense. We did not mean that self-assembled monolayers (SAMs), silanes, or other primer layers would become irrelevant. Rather, they often remain useful for installing azide, alkyne, tetrazine, or alkene handles on electrode surfaces. On the other hand, the point is that wearable-biosensor performance should not be determined by self-assembly or non-selective covalent coupling alone at the decisive step of bioreceptor immobilization. Classical approaches, including physisorption, SAM-guided passive presentation, and EDC/NHS coupling, frequently leave unresolved problems of random receptor orientation, hydrolytically vulnerable attachment, incomplete or variable loading, and poor transferability to deformable carbon, polymeric, and textile substrates. Bioorthogonal click chemistry moves the interface beyond these limitations by converting pre-installed surface handles into chemically defined covalent junctions with controlled stoichiometry, minimal off-target reactivity, and application-specific kinetic and biocompatibility profiles. Accordingly, each reaction class in this review is evaluated against the specific failure mode it best resolves: CuAAC for quantitative and reproducible surface loading, SPAAC for copper-free immobilization of fragile biomolecules, IEDDA for ultrafast catalyst-free ligation at low reagent doses, and thiol-ene/yne photoclick chemistry for patterned covalent functionalization of soft wearable substrates.

An important adjacent field outside the primary scope of this review is wearable colorimetric sensing. Recent reviews show that visible-readout and microfluidic colorimetric patches now form a major branch of epidermal diagnostics, offering low-cost, simply fabricated, and naked-eye or smartphone-based readouts for sweat biomarkers, including glucose, lactate, chloride, urea, and pH [26,27,28,29,30]. Dedicated devices can operate with only a few microliters of sweat and are highly attractive options for disposable, visually interpretable screening [29]. At the same time, the same studies make clear that colorimetric wearables face a different set of limitations from electrochemical systems: quantitative accuracy depends on image capture and lighting conditions; many chromogenic reactions are irreversible or semiquantitative; continuous or closed-loop operation typically requires elaborate chrono-sampling microfluidics because the readout is not intrinsically reversible. For these reasons, the present review focuses on electrochemical sensors rather than colorimetric patches. In the concept of this study, the main aim is not to dismiss colorimetry; rather, the aim is to explain why more elaborate interface engineering (including click-defined bioconjugation) becomes a worthwhile investment when the application requires electronically integrated, quantitatively resolved, continuous or repeatedly sampled monitoring with tight control over drift and multiplexed signal routing rather than simple episodic visual readout.

## 2. The Chemist’s Toolkit: Tailoring “Click” Reactions for Electrodes

The major click reactions discussed below are not presented as interchangeable surface ligations, but as solutions to different shortcomings of classical immobilization chemistry. CuAAC, SPAAC, IEDDA, and thiol-ene/yne photoclick reactions all produce covalent interfaces. However, they differ in the way they address the central wearable biointerface problems of orientation control, bond stability, reproducibility, catalyst compatibility, and substrate adaptability [31,32,33]. Accordingly, each subsection below follows the same comparative logic, asking the following questions: What problem remains unresolved by physisorption, SAM-guided passive presentation, or EDC/NHS coupling in this context? Which mechanistic feature of the click reaction resolves that problem? What trade-offs or residual limitations remain? The value of click chemistry, therefore, lies not merely in forming covalent bonds but in enabling deterministic and application-specific interface engineering. These advantages are not unconditional; in practice, catalyst management, handle accessibility, surface heterogeneity, and manufacturing simplicity often determine whether a given click reaction is genuinely superior to a conventional immobilization route. Therefore, the chemist’s toolkit of click reactions offers guidance, not only in selecting a reaction class, but also in designing the covalent junction that will ultimately govern steric burden, bond stability, and electron-transfer behavior at the interface. Accordingly, Table 1 compares the major click reactions at both the reaction level and the product level, because the character of the formed linkage is more informative for biosensor design than a simplified mechanism cartoon.

### 2.1. Cu(I)-Catalyzed Azide–Alkyne Cycloaddition (CuAAC)

**Figure 2:**
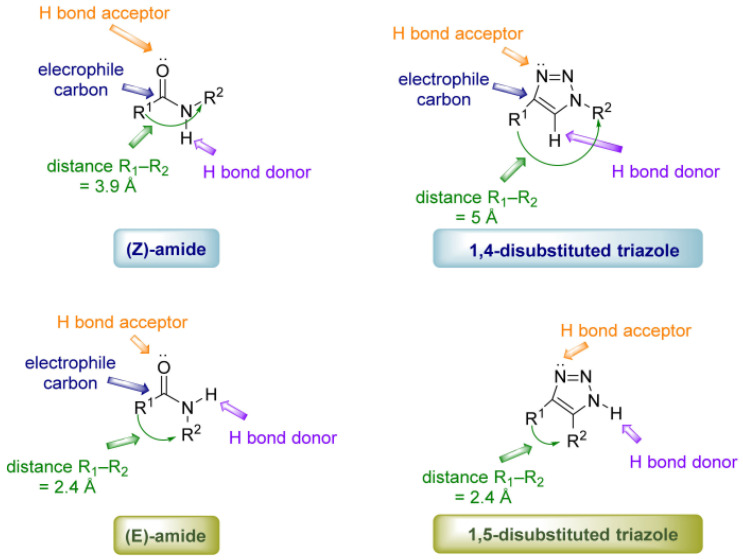
Demonstration of superimposition of the 1,2,3-triazole moiety and a native *trans*-peptide bond. The similarities in terms of topology and electronic behavior lead to the triazole linkage for emulating the amide bond geometry [39].

Even when classical surface chemistry provides an ordered primer layer, the decisive biomolecule-coupling step often remains variable if it relies on passive adsorption or EDC/NHS chemistry. CuAAC moves the interface beyond self-assembly by converting azide- or alkyne-presenting surfaces into triazole-linked bioreceptor layers with near-quantitative coupling and controllable loading, with the potential for improved batch reproducibility when the precursor interface is itself well-defined. Its comparative advantage is therefore most evident when dense, electronically well-defined, and mechanically stable interfaces are required; this relies on the condition that copper can be rigorously complexed, removed, and kept away from sensitive biological components. CuAAC, often hailed as the “gold-standard” click reaction, is the prototypical 1,3-dipolar cycloaddition between an azide and a terminal alkyne catalyzed by Cu(I) to form a 1,2,3-triazole linkage [38]. Specifically, Cu(I) coordinates to the alkyne, activating it toward nucleophilic attack. It is conducted by the azide, which stabilizes the metallacyclic transition state, ending up at the 1,4-disubstituted triazole isomer. The reaction was first introduced by Sharpless et al. in 2001 [16], exemplifying the click philosophy. Therefore, the technique became widely recognized, since it provides high yields and strong regioselectivity with no byproducts. The reaction forms a triazole linkage that is highly stable and chemically inert, which is comparable to an amide bond, which is often more resistant to hydrolysis. In wearable sensor applications, CuAAC enables site-specific and covalent attachment of bioreceptors by avoiding many limitations of conventional random immobilization strategies [39,40,41], as demonstrated in Figure 2. For example, an azide handle can be utilized in a predefined position on a protein or aptamer by benefiting from amino acid mutagenesis or selective chemical modification. This enables the coupling of the biomolecule with an alkyne-functionalized electrode at the desired site. The reaction allows preserving the intended biorecognition orientation. Thus, site-directed immobilization of the biorecognition elements dramatically enhances the electron transfer rate and improves the analyte accessibility on the electrode surface [42,43].

The main advantages of the CuAAC reaction for electrode functionalization can be specified as the speed and yield, supported by a broad substrate scope, and tolerance of ambient aqueous environments. The reaction can be completed at room temperature in minutes to hours, and its rate is ~10^7^-fold higher than the uncatalyzed azide–alkyne cycloaddition. Such kinetics facilitate high-density monolayer formation on electrodes, since even densely packed azides and alkynes will react to completion given a sufficient Cu(I) catalyst supply. The triazole linkage formed confers mechanical and chemical stability to the biointerface. Unlike labile silane or amide bonds, triazoles do not hydrolyze or rearrange, even under prolonged exposure to biofluids or sweat. Hence, covalent triazole-linked films show far greater resistance to delamination under shear stress than classic adsorption or EDC/NHS-coupled layers [44]. Another advantage is orthogonality, as azides and alkynes are absent in natural biomolecules, so nonspecific reactions are minimal in complex samples. The CuAAC can thus be carried out in complex media or on biomolecule-functionalized surfaces without off-target side reactions. CuAAC is widely used because it can functionalize many different materials. It has been applied by Yáñez et al. to electrode surfaces, nanomaterials, metallophthalocyanines, and polymers with a variety of biosensor systems. This enables the stable attachment of enzymes, antibodies, nucleic acids, and other biomolecules to these platforms. This includes conventional gold electrodes, carbon electrodes, carbon nanotubes, graphene, and even conductive polymers [38]. The common strategy for the gold electrodes is to form a self-assembled monolayer (SAM) by including a clickable functional group, then perform CuAAC with the complementary group on the target biomolecule. For instance, Collman et al. demonstrated an azide-terminated alkanethiol SAM on Au that was efficiently clicked with various alkynes in the presence of Cu(I), covalently functionalizing the surface. Conversely, alkyne-terminated thiols can be self-assembled and reacted with azide-bearing molecules. The thiol–Au chemistry, as a coordinated covalent bond, is used in the click handle to the surface. When the click reaction takes place, triazole formation leads to the locking of the bioreceptor in on the electrode surface. To improve monolayer packing and control orientation, mixed self-assembled monolayers (SAMs) are often used. For example, azidoundecanethiol can be diluted with an inert thiol (such as octanethiol). This produces a well-ordered azide-terminated monolayer, reduces steric crowding, and enables near-complete conversion to the triazole once an alkynyl ligand is introduced. In contrast, carbon electrodes such as glassy carbon, screen-printed carbon, graphene, and carbon nanotubes do not support thiol-based anchoring. In these cases, CuAAC is typically enabled by first attaching azide or alkyne groups to the surface via covalent grafting. A powerful method is the electrochemical reduction in aryl diazonium salts bearing an azide functionality, which forms a robust aryl monolayer on carbon surfaces [38]. The electrode-bound azide can then undergo CuAAC with an alkyne-terminated molecule of interest. This two-step grafting, then clicking approach has been used to tether ferrocene redox reporters, DNA oligonucleotides, and even entire nanoparticles to carbon electrodes. Similarly, single-wall carbon nanotubes functionalized with alkyne groups on their sidewalls were clicked to azide-tagged proteins (cytochrome *b*562 in one case), achieving stable protein–nanotube conjugates for biosensing. In another example, multi-walled CNTs were decorated with azide-functional β-cyclodextrins and clicked with alkynylated polymers to create nanocomposites for sensors. Graphene surfaces, while atomically smooth, can be click-functionalized through defect sites or edge chemistries: for instance, graphene sheets were modified with aryl azides via diazonium chemistry and then coupled to alkyne-terminated gold nanoparticles, creating hybrid graphene–Au structures. The click reaction’s specificity ensured the graphene lattice remained intact while the nanoparticles were tightly attached, enabling enhanced electrochemical detection. Finally, polymer and textile electrodes (such as conducting polymer films or fibers) can be functionalized by incorporating click-reactive groups into the material. One strategy is to electropolymerize a monomer that carries an azide or alkyne pendant, producing a conductive film ready for click coupling [38]. In the studies reported by Bu and Scavetta demonstrated that the electrodeposition of azidomethyl-functionalized PEDOT (PEDOT-N_3_), which was clicked with ethynylferrocene via CuAAC, resulted in a ferrocene-decorated film. The surface was used in the detection of dopamine with an amperometric method at low micromolar levels [45,46].

Cu(I) catalyst requirement defines the CuAAC reaction in essence. However, it can also be a drawback in biosensor construction processes. Normally, copper is a concerning metal in biological settings, since it is cytotoxic and can rapidly inactivate proteins via binding to residues like histidine, and can also promote oxidative damage. Moreover, any residual copper ions in the wearable sensors integrated on the skin or interstitial fluid gradually harm nearby cells or tissues. Copper can also interfere with electrochemical readouts since it has a specific oxidation behavior at a specific potential. Therefore, if copper is not fully removed, the trace of the element on the electrode may cause the generation of undesired redox currents like Cu(II)/Cu(I) peaks or catalyze side reactions that raise the background signal [47]. Thus, several strategies have been developed in order to overcome the issues. One of the most utilized approaches is to use accelerating ligands to chelate Cu(I), such as tris(triazolylmethyl)amine derivatives (e.g., TBTA, THPTA, and BTTAA). These ligands not only speed up the click reaction and sequester copper but also minimize the possible exposure to free ions [48]. Therefore, the high rate of click reaction can be obtained by using a lower (sub-millimolar) amount of copper concentration. It helps in reducing toxicity and simplifies post-reaction cleanup. Another elegant strategy in electrochemistry is the electro-click method. Instead of adding Cu(I) salts in bulk solution, Cu(II) is added and electrochemically reduced in situ on the electrode surface to generate the active Cu(I) catalyst locally [48,49,50]. Devaraj and co-workers demonstrated that CuAAC can be electrochemically gated by reducing an inactive Cu(II) complex to the catalytically active Cu(I) state at the electrode (reported at ca. −300 mV vs. Ag/AgCl), enabling localized, addressable surface coupling and minimizing cross-contamination on multielectrode platforms. Surface coverage can be tuned by the applied bias and reaction time, and the electrode can be rinsed after coupling to remove residual copper. In general, CuAAC proceeds under mild conditions in buffered aqueous media and can tolerate mixed aqueous–organic co-solvents (e.g., DMSO or alcohols) when hydrophobic reagents are used, typically at room or physiological temperature and near-neutral pH. Because dissolved oxygen can oxidize Cu(I) to Cu(II) and slow the reaction, degassing and/or using sodium ascorbate (often with suitable Cu-stabilizing ligands) is commonly employed to maintain an effective Cu(I) catalyst concentration. The high packing density of uniformly oriented receptors increases signal amplitudes (more redox labels or enzyme activity per area) and can lower the limit of detection. The rigid triazole linkage also improves electron transfer kinetics by ensuring conjugation in some cases (e.g., attaching conjugated linkers via triazoles) and by preventing insulating movements of the biomolecule. Studies comparing CuAAC-immobilized biosensors to those made with random thiol self-assembly or EDC coupling have found the click-functionalized interfaces to be more reproducible. It lowers batch-to-batch variation and retains activity longer in real-life wear conditions. The primary limitation of CuAAC for wearable devices remains the catalyst [51]. From a translational standpoint, however, CuAAC should not be treated as automatically simple or low-cost. Reliable implementation on biomolecules and complex electrode interfaces often requires not only Cu salts, but also reductants, oxygen management, and Cu(I)-stabilizing ligands such as THPTA or BTTAA, which add reagent burden, cleanup steps, and analytical quality-control requirements for residual copper. A second practical issue is that the near-quantitative conversions often demonstrated on flat, well-ordered gold SAMs cannot be assumed for rough, porous, or chemically heterogeneous wearable electrodes [52]. On screen-printed carbons, CNT papers, graphene composites, and polymer-coated electrodes, local variability in handle density, diffusion, and surface accessibility can produce incomplete or spatially nonuniform clicking across the real electroactive surface. In such cases, CuAAC remains highly valuable, but its practical superiority depends on whether the gain in surface definition justifies the added catalyst management and validation burden. Therefore, claims of “quantitative” CuAAC immobilization are most convincing when supported by surface coverage or functional conversion measurements on the actual electrode architecture, together with residual copper analysis after washing.

### 2.2. Strain-Promoted Azide–Alkyne Cycloaddition (SPAAC)

Strain-promoted azide–alkyne cycloaddition (SPAAC) addresses the same core weaknesses of classical immobilization, random orientation, poor long-term stability, and incomplete loading, but solves an additional problem that CuAAC may introduce in wearable systems: exposure to copper. Relative to EDC/NHS or passive adsorption, SPAAC still provides site-selective, triazole-forming covalent fixation; relative to CuAAC, it preserves biomolecule activity and simplifies processing by eliminating catalytic metal. Its strongest use case is therefore fragile, copper-sensitive, or in vivo-adjacent biointerfaces where deterministic immobilization is required under mild conditions. SPAAC is a copper-free variant of the triazole-forming click reaction, relying on a ring-strained cyclooctyne (a cyclic alkyne) to react directly with an azide [53]. In SPAAC, the substantial ring strain (≈18 kcal/mol) of cyclooctyne derivatives drives the azide addition without the need for a metal catalyst. The mechanism is analogous to CuAAC (a 1,3-dipolar cycloaddition yielding a triazole), except that ring strain (and, in some cases, electronic activation) lowers the activation barrier rather than Cu(I) coordination. Early and widely used SPAAC cyclooctynes include DIFO and BARAC (Bertozzi and co-workers), as well as BCN and aza-dibenzocyclooctyne derivatives such as DIBAC/ADIBO (van Delft/Rutjes and co-workers), alongside dibenzocyclooctyne scaffolds (DBCO/DIBO) developed in parallel by other groups [54,55]. The mentioned compounds demonstrate a rigid cycloalkyne structure, which is mostly reinforced with electron-withdrawing fluorine or aromatic substituents to further increase reactivity. For instance, DBCO is a widely used SPAAC reagent that has a strained dibenzocyclooctyne core showing the capability of undergoing cycloaddition with azides rapidly at room temperature, forming the same 1,2,3-triazole linkage as CuAAC [56]. Because no external catalyst is required, SPAAC is considered fully bioorthogonal and biocompatible by its unique design. Thus, it proceeds in living systems without perturbing native biomolecules, which was a key motivation for its development. The main advantage of SPAAC for wearable biosensors is the elimination of copper, thereby avoiding the toxicity, enzyme inhibition, and electrode fouling issues associated with CuAAC’s catalyst [54,57]. This makes SPAAC ideal for in vivo or in situ bioconjugation scenarios. For instance, attaching a bioreceptor to a device after it has been applied to the body, or functionalizing surfaces that will contact fragile biological fluids or cells. Therefore, Kim et al. emphasize that copper-free click chemistries like SPAAC have enabled numerous biomedical applications in vitro and in vivo that would be impractical with CuAAC. In the context of sensor fabrication, SPAAC allows one to perform the immobilization in one step under physiological conditions at pH~7.4, 37 °C, and in aqueous buffer without additional reagents. This simplicity not only reduces processing steps (no need for degassing or adding reductants/ligands) but also maintains biomolecule activity. Thus, delicate enzymes or antibodies retain their structure because they are not exposed to copper or harsh chemicals during attachment.

Another advantage of SPAAC is that its reagents can be engineered for better performance. It is known that CuAAC is limited only to terminal alkynes. On the other hand, the SPAAC method enables access to a variety of cyclooctynes. These cyclooctynes enable the researchers to increase the speed and physical properties of the reaction. For example, BARAC has electron-withdrawing carbonyl groups that enable azide addition. This addition leads to second-order rate constants between 1–3 M^−1^/s, which is much faster than earlier DBCO reagents, which had rates of 0.2–0.5 M^−1^/s. Some of the cyclooctynes are also utilized in hydrophilic chains to improve their solubility in water, such as PEGylated DBCO. This enhancement may boost the effective local concentration of the reagent at hydrophobic surfaces. Ultimately, this tunability enables surface chemists to choose a cyclooctyne derivative tailored for a specific sensor platform. The proper decision shall strictly depend on whether the focus is on reaction speed, stability, or solubility [58]. When it comes to the surface stability perspective, SPAAC produces the same triazole bond as CuAAC. That bond has a strong resistance to hydrolysis and mechanical stress. However, the main difference between these is how each reaction condition affects the film or layer underneath. Because SPAAC happens under mild conditions, usually without a catalyst or heating, it typically better maintains delicate surface chemistry [53].

Despite the mentioned advantages and speed, SPAAC also has some limitations in terms of electrode functionalization. It can be specified mainly related to its slower kinetics and the steric bulk of its cyclooctyne reagents. The reaction rate of SPAAC is still modest compared to that of CuAAC, even though the orders of magnitude faster than a non-strained cycloaddition [16,59]. Typical second-order rate constants are in the order of 10^−2^–10^0^ M^−1^s^−1^ (depending on the cyclooctyne). This means that reaction times of 1–12 h may be needed to achieve high coverage, especially at the low (micromolar) concentrations of bioreceptor often used. For a densely azide-functionalized electrode, this prolonged reaction time could allow competing processes (nonspecific adsorption of other biomolecules, or azide hydrolysis in rare cases) if done in complex media. In practice, SPAAC coupling yields on surfaces are high, but incomplete conversion is possible if the surface azide density is very high and cyclooctyne cannot access every site due to steric hindrance. The bulky substituents on reagents like DIBO or DBCO (which have several aromatic rings) can sterically exclude neighboring azides from reacting if the spacing is too tight [60]. This is one reason mixed-monolayer strategies are also valuable for SPAAC: diluting azide SAMs with inert spacers (an azide–alkanethiol mix on gold) provides the cyclooctyne with enough room to approach each azide and form the triazole. Another practical limitation is the cost and storage stability of cyclooctyne reagents. Compared to regular terminal alkynes or azides, cyclooctynes are more complex to synthesize and usually more expensive. This is an important factor for large-scale sensor manufacturing. Additionally, these reagents have a limited shelf life. Several reports indicate that some BCN derivatives can experience slow oxidative degradation or polymerization while stored. Therefore, it is best to use freshly prepared materials when possible or to verify the integrity and reactivity of the reagents, for example, through a quick test coupling, before important conjugation steps. Lastly, the steric effects of the surface coating should also be considered, as they can greatly affect coupling efficiency and overall functionalization density. The study by Park et al. found that the choice of linker connecting the azide to a surface significantly impacted whether SPAAC would reach completion or cause film disruption [53,61]. A short, hydrophobic alkyl linker on a tyrosine-azide resulted in a tightly cross-linked polyphenolic film that remained intact during SPAAC. In contrast, a longer, more hydrophilic PEG linker caused film swelling and partial delamination when exposed to bulky DBCO reagents. This suggests that surfaces need to be designed to handle the structural changes caused by cycloaddition by using cross-linked or strongly adsorbed primer layers, especially on smooth, non-porous electrodes. Finally, while not a chemical limitation, the relatively slow rate of SPAAC means it is not ideal for real-time sensing reactions. Instead, SPAAC is typically used in the fabrication phase of the sensor (e.g., attaching an antibody to the electrode before use) rather than during the sensing operation itself.

Implementing SPAAC on different electrode materials is conceptually straightforward since it mirrors the CuAAC approaches minus the catalyst. On gold, one popular route is to form an azide-terminated SAM and then incubate the electrode in a solution of a cyclooctyne-bearing biomolecule [56]. Azidoundecanethiol SAMs on Au have been reacted with DBCO-functionalized DNA and proteins to achieve site-specific immobilization without copper, yielding monolayers of comparable quality to CuAAC-derived ones. Alternatively, one can assemble the cyclooctyne on the surface and have azides in solution. For example, alkanethiols presenting a cyclooctyne (such as an ADIBO-thiol) can form a SAM, which is then exposed to azide-labelled biomolecules. This approach has been used to orient antibodies site-specifically, where antibodies engineered with a single azide (through azidohomoalanine or glycans) were SPAAC-coupled to a cyclooctyne SAM on gold, achieving oriented monolayers that improved antigen binding by >50% compared to random attachment. On carbon and graphene surfaces, SPAAC requires an analogous “primer” step as CuAAC, typically attaching an azide functionality to the carbon via covalent chemistry. A common method is still aryl diazonium grafting (4-azidobenzene diazonium on a CNT or graphene electrode) to present azides densely on the surface [62,63]. The carbon electrode is then simply dipped in a solution of the cyclooctyne-modified ligand. Because no catalyst or electrochemical step is needed, this process is experimentally simple and amenable to flexible substrates and screen-printed carbon inks, where applying uniform potential or heat might be difficult. There are reports of graphene oxide (which has residual alkene groups) being directly functionalized via SPAAC as well; however, generally, attaching a well-defined azide or alkyne to graphene first yields more reproducible results. For polymer electrodes and hydrogels, SPAAC offers an excellent route to biofunctionalization since many polymers can be synthesized or post-modified to carry azide groups. For instance, a conductive polymer like PEDOT:PSS can be chemically derivatized to add azide sidechains (PEDOT-N_3_ forming a clickable film [23]. Fenoy et al. showed that such PEDOT-N_3_ films in organic electrochemical transistors could be functionalized by both CuAAC and SPAAC to attach biomolecular recognition elements. In one case, they clicked a poly-L-lysine polymer bearing multiple DBCO groups onto a PEDOT-N_3_ channel, creating a dense network of DBCO on the device surface for subsequent protein immobilization [23]. This two-stage use of SPAAC (first to attach a polymer linker, then to attach a biotin and capture proteins) highlights its utility in building multilayer architectures on polymer substrates. Crucially, SPAAC can also be performed on flexible or large-area surfaces (like textile fibers coated with nanocarbon or polymer). Since no electrical input or heating is required, one can batch-immersion functionalize many electrodes at once. The mild conditions also mean that polymer substrates, including PET and PDMS, will not be chemically attacked. The plasma-activated PDMS surface decorated with azides can undergo SPAAC to attach DNA probes, for example, without the PDMS degrading, whereas CuSO_4_/ascorbate might slowly etch or swell it. Additionally, SPAAC has been successfully employed in situ on living cell membranes and tissue surfaces. Moreover, one can imagine future wearable devices that directly SPAAC-couple to biomolecules on the skin or in biofluids for ultra-integrated sensing, something not feasible with copper catalysis.

SPAAC’s performance on electrodes is influenced by factors such as reagent concentration, solvent, and steric environment rather than external triggers, since chemically it needs no catalyst or energy input. Concentration is often the deciding factor for coupling yield, as using a higher concentration of the cyclooctyne in solution drives the surface reaction closer to completion in a reasonable time. However, cyclooctyne reagents are often used at tens of micromolars to avoid waste due to cost and to conserve precious biomolecules (like DBCO–antibody conjugates). At such concentrations, reaction times of several hours at room temperature are typical to achieve near-saturation of surface azides.

Gentle heating (37 °C for proteins that tolerate it) can modestly accelerate SPAAC, roughly doubling the rate for a 10–15 °C increase, but often it is unnecessary. The solvent composition can also play a role in SPAAC between a hydrophobic cyclooctyne (like DBCO) and a hydrophilic azide on a surface and may benefit from the addition of ~5–20% organic cosolvent (DMSO or DMF) to help solubilize the cyclooctyne [64,65,66]. However, too much organic solvent can collapse SAMs or dehydrate polymer films, so typically a predominantly aqueous buffer with a small cosolvent percentage is optimal. Steric and strain effects of the reagents themselves have been mentioned by using a smaller cyclooctyne (such as BCN, which lacks bulky aromatics) can improve access to crowded surface azides. A clever way to diminish steric hindrance is to employ a spacer on one of the reactants. Using an azide–PEG–SAM on gold will space the azide away from the surface and provide flexibility to better capture a cyclooctyne. Conversely, using a cyclooctyne that includes a short PEG spacer before its reactive alkyne can help it thread into densely packed azide layers (some commercial DBCO reagents have PEG4 linkers attached for this reason). Reaction monitoring on surfaces can be done electrochemically if one of the components carries a redox label. For example, CuAAC attachment of an ethynyl–ferrocene probe to an azide-terminated monolayer produces a surface-confined ferrocene voltammetric signal, enabling electrochemical tracking of surface reaction progress [67]. Such measurements show that most of the coupling happens in the first 1–2 h for moderate-density monolayers, then slowly approaches a plateau, which is consistent with a pseudo-first-order process that slows as surface sites become scarce. SPAAC-derived interfaces generally exhibit excellent biocompatibility and stability, akin to CuAAC interfaces. Notably, the absence of copper means that enzymes immobilized via SPAAC retain higher activity (copper can sometimes inhibit enzyme active sites even if present transiently). In one report, a glucose oxidase clicked to a carbon nanotube electrode via SPAAC retained ~90% of its solution-phase activity, compared to ~50–60% when attached by conventional carbodiimide chemistry, attributed to the benign, fast coupling which does not perturb the enzyme. The triazole linkage from SPAAC also yields a more stable baseline in sensing.

Electrodes functionalized by SPAAC showed negligible drift over days of continuous operation in fluid, whereas analogous sensors using physical adsorption lost significant signal due to protein desorption. The high specificity of SPAAC means that surface fouling is minimized, since the cyclooctyne will not react with common fouling agents (proteins, salts, etc.), and one can functionalize even in complex media. This was highlighted by a recent demonstration of SPAAC on actual biological tissue, where azide-functional hydrogel patches were SPAAC-linked to DBCO-modified antibodies in human serum, achieving the intended bioconjugation without any noticeable interference from serum components. For wearable sensors that operate in biofluids like sweat or blood, such selectivity is crucial. In summary, SPAAC provides a powerful copper-free method to functionalize electrodes with biomolecules in a controlled, biocompatible manner. Its slower kinetics and bulkier reagents require thoughtful surface and reaction design by using spacers, choosing optimal cyclooctynes, and allowing sufficient reaction time. However, when optimized, SPAAC yields interfaces with high bioactivity, stability, and low background, all essential for reliable wearable biosensing.

### 2.3. Inverse Electron-Demand Diels–Alder (IEDDA) Cycloaddition

**Figure 3:**
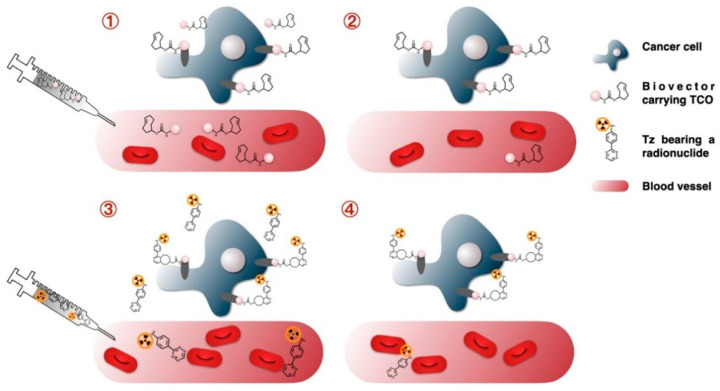
The overall mechanism of the Inverse Electron Demand Diels–Alder (IEDDA) reaction is the cycloaddition between a tetrazine derivative and trans-cyclooctene (TCO). (1) refers to fast reaction rate, (2) stands for chemoselectivity, (3) is for no interference with the living system, and (4) represents no toxicity. The irreversible release of nitrogen gas (N_2_) causes the reaction to proceed forward, enabling ultrafast kinetics that approach the diffusion limit [70].

The inverse electron-demand Diels–Alder (IEDDA) approach becomes especially valuable when the limitation of classical coupling is not only instability but also reaction speed. Slow immobilization can allow dilute or fragile bioreceptors to denature, adsorb nonspecifically, or be wasted before efficient surface capture occurs. By combining catalyst-free conditions with exceptionally fast tetrazine-strained alkene ligation, IEDDA moves beyond classical surface assembly toward rapid, low-dose, and highly selective covalent fixation. It is therefore particularly attractive for time-sensitive immobilization, low-concentration probes, and orthogonal multiplexing strategies. The IEDDA reaction between 1,2,4,5-tetrazines and strained alkenes has quickly become one of the most effective bioorthogonal click reactions. This is mainly because of its unusually fast kinetics, although its practical adoption depends on handle stability, accessibility, and whether that kinetic advantage solves a real fabrication bottleneck. In this [4 + 2] cycloaddition, an electron-deficient diene (the tetrazine) reacts with an electron-rich dienophile, typically trans-cyclooctene (TCO) or similar strained alkene structures. This reaction produces a dihydropyridazine product while releasing N_2_ gas [68]. The process is driven by the relief of ring strain in the alkene and the quick elimination of nitrogen, which makes the overall reaction effectively irreversible. Mechanistically, the reaction starts with a Diels–Alder cycloaddition that forms a bicyclic intermediate. This is followed by a retro Diels–Alder step that releases N2 and creates a stable, covalently linked adduct, usually a substituted dihydropyridazine. Importantly, the reaction does not require a catalyst or any external trigger. It can occur spontaneously in water and even in living systems, showing its bioorthogonality and wide compatibility. For the fastest combinations of tetrazine and TCO, second-order rate constants are reported to reach around 10^5^–10^6^ M^−1^/s, which is close to the diffusion limit in water [69]. These ultra-fast kinetics allow for efficient bioconjugation at micromolar and even nanomolar reactant concentrations. This feature is especially useful for sensor functionalization, where only small amounts of expensive bioreceptors might be available [70] (Figure 3).

The IEDDA reaction offers several important advantages for electrode functionalization, but these must be weighed against reagent stability, handle-installation complexity, and cost. First, the reaction is extremely fast and can be completed within seconds–minutes. This is particularly advantageous when modifying sensor surfaces with fragile biomolecules, since the brief exposure to the reagents minimizes the time biomolecules spend in potentially non-ideal conditions [71,72]. For instance, antibody immobilization via tetrazine–TCO can essentially be a mix-and-done step, locking the antibody onto the surface before it has time to denature or aggregate in solution. The rapid kinetics also mean that very low concentrations of capture agents can be efficiently attached. A dilute (nM) solution of a TCO-modified aptamer will still rapidly conjugate to a tetrazine-coated electrode, whereas slower SPAAC or CuAAC might not yield appreciable coupling at such low concentration within practical timeframes [70]. This efficiency at low concentration is crucial when dealing with precious or limited-supply bioreceptors, including aptamers arising from small-scale synthesis or antibodies from a limited hybridoma batch. Additionally, the absence of any catalyst or harsh reagents places IEDDA on par with SPAAC in terms of biocompatibility, no metal ions, and no aggressive chemicals. Both tetrazines and trans-cyclooctenes are generally nontoxic at the micromolar levels used and have been employed in vivo for imaging tumors in mice and humans with minimal side effects [73]. For wearable sensors intended for direct skin contact or implantable use, this means one could conceivably perform an IEDDA conjugation on the device in situ. For example, clicking a tetrazine-functional hydrogel sensor to a TCO-modified targeting ligand on the skin. Another advantage is the chemoselectivity of the IEDDA, since tetrazines are very selective for strained dienophiles. While they can react with unstrained alkenes (like those in certain amino acids or lipids), the rates for those reactions are many orders of magnitude lower than with a trans-cyclooctene. Thus, in complex biological fluids, a tetrazine on a surface will preferentially find and react with a TCO label rather than be quenched by random biomolecules. This selectivity is governed by the widespread use of tetrazine probes in live-cell fluorescence tagging, since endogenous unsaturated compounds typically do not interfere appreciably [74]. For sensor surfaces, this implies that a tetrazine-coated electrode can be introduced into a biological sample containing a TCO-tagged analyte or secondary molecule, and the click will occur cleanly without nonspecific bindings. From a stability standpoint, the pyridazine linkage formed by IEDDA is very robust. It is essentially an aromatic or partially aromatic ring system that is not prone to hydrolysis or photolysis. Studies on tetrazine–ligation bioconjugates have shown they remain stable in serum and inside cells for several days. Once an electrode is functionalized through IEDDA, the covalent bond is not likely to be the main weak point for long-term performance. Instead, other surface components may be more at risk. This includes the self-assembled monolayer (SAM) or polymer layer, which might break down first. IEDDA works well for modular surface engineering. Tetrazine or TCO handles can easily fit into many types of scaffolds, such as polymers, dendrimers, and nanoparticles, and then bond with corresponding surface functionalities. For instance, a dendrimer with a tetrazine end can link to a surface that presents TCO in a quick, single step. This allows for the multivalent display of functional groups and possibly several copies of a bioreceptor. Such multivalent connections can boost the number of binding sites in a given area, which enhances sensor responses and improves analyte capture efficiency.

Despite its many strengths, the IEDDA ligation has some practical limitations for electrode functionalization. One consideration is the stability of the reactants, where tetrazines are somewhat sensitive compounds. Many 3,6-disubstituted s-tetrazines, which are the most common type used for bioorthogonal reactions, are prone to gradual hydrolysis or oxidation, especially in aqueous solution or upon exposure to light. They typically have a limited shelf life in solution in the order of days–a couple of weeks at 4 °C, and longer in lyophilized form. Similarly, trans-cyclooctenes can undergo slow isomerization to the inert cis-cyclooctene (with half-lives ranging from days to months depending on substitution) [70]. They can also react with themselves in certain cases by dimerization via Diels–Alder with a second TCO, although this is usually negligible at low concentrations. For sensor fabrication, this means that one should use freshly prepared tetrazine/TCO reagents and avoid prolonged storage of surfaces decorated with these groups before clicking. A tetrazine-functionalized electrode might lose some reactivity over time if exposed to ambient conditions, as tetrazine groups could decompose [75,76,77,78]. The electrodes can be stored in a desiccated form in the dark, or the click step can be performed immediately after surface functionalization with tetrazine in order to slow this process.

Another limitation is the potential side reactivity of tetrazines with certain electron-rich aromatic compounds. Tetrazines can undergo IEDDA with strained alkynes like cyclooctynes, though at lower rates than with TCO [68], and even engage in pericyclic reactions with highly electron-rich aryl systems, which is the basis of some small-molecule probes but is rarely an issue in biosensing. Particularly, it must be ensured that there are no unintended strained bonds on the surface or analyte. For instance, if a polymer coating on the electrode contains pendant norbornene groups, a tetrazine might react with it. Thus, one must avoid mixing IEDDA handles with other click handles unintentionally. The fast reaction rate, while generally an advantage, means that if both reactive partners are present in solution, they will click together before one can be attached to the surface. This necessitates a sequential approach, where one component must be immobilized first, and then the other is introduced. For example, a tetrazine-functionalized protein and a TCO-functionalized surface in one pot cannot be mixed with other molecules present. Therefore, one must first attach the tetrazine-protein to the surface or vice versa. This is not a drawback, but it does require that the conjugation sequence be planned in sensor assembly. Another consideration is that tetrazine ligations often produce a distinct optical signature, since tetrazines are typically purple-colored and fluorescently quenched, but upon reaction, their product is colorless and may fluoresce. On a sensor surface, a high density of tetrazine could potentially absorb light. However, this is usually negligible for electrochemical sensors, and it can even be exploited by visually verifying a reaction by the loss of tetrazine’s color on the surface. Finally, both tetrazine and TCO derivatives are more specialized than standard azide or terminal alkyne handles, and their use introduces a nontrivial practical burden in terms of procurement, synthesis, purification, and storage. Although synthetic access to both TCOs and modern tetrazine scaffolds is improving, these handles remain less straightforward for routine adoption than simple azides or alkynes [79]. Therefore, the exceptional kinetics of IEDDA should not be interpreted as an automatic practical advantage for all wearable-biosensor workflows. In routine ex situ fabrication steps, where immobilization can proceed over tens of minutes or hours, a much faster ligation does not necessarily translate into a better or more economical manufacturing process. The premium associated with tetrazine/TCO chemistry is most justified when one or more of the following conditions apply. The bioreceptor is available only at very low concentrations; exposure time must be minimized to preserve activity. Catalyst-free rapid ligation is required directly on-device or in situ, or orthogonal multiplexing requires a dedicated tetrazine/TCO channel. By contrast, for batch fabrication of disposable sensors with stable biomolecules and no time-critical assembly step, SPAAC or CuAAC may provide a more favorable cost-to-benefit balance. IEDDA should therefore be viewed not as a universally superior option, but as a specialized high-value solution when reaction speed, low-dose efficiency, or orthogonality clearly outweighs handle cost and procurement complexity.

Implementing IEDDA click chemistry on electrodes involves immobilizing one of the two reactive partners (tetrazine or the strained alkene) on the surface. Both approaches have been demonstrated. A common strategy is to put the tetrazine on the electrode, since tetrazines are small (<300 Da in many cases) and can be easily integrated into surface chemistry schemes. For instance, a carboxylic acid- or amine-terminated SAM on gold can be used to attach a tetrazine by standard NHS-ester or EDC coupling, which is one case where a classical coupling is acceptable to introduce the click handle. The result is a tetrazine-presenting monolayer, ready to capture any TCO-functionalized biomolecule in solution. Alternatively, a thiol-functionalized tetrazine could be synthesized for direct SAM formation on gold, though in practice, the stability of tetrazines under the conditions of SAM assembly must be ensured. On carbon surfaces, aryl diazonium or silane chemistry can be used to attach tetrazines. For example, 4-(2,3,5,6-tetrazine)phenyl diazonium salts have been used to graft tetrazines onto glassy carbon and carbon nanotubes, yielding clickable carbon electrodes that react with TCO probes.

Another approach is to first coat the electrode with a polymer or coating that has built-in tetrazine groups. A recent work by Hasler et al. described clickable graphene nanoribbons where graphene nanoribbon films were functionalized along their edges with tetrazine moieties. These tetrazine-bearing graphene interfaces could then be conjugated with TCO-containing biomolecules, combining the electrical advantages of graphene with the bioconjugation specificity of IEDDA. On the other hand, one can immobilize the TCO as a strained alkene on the surface and have tetrazine in solution. This is slightly less common because many TCO derivatives are hydrophobic or need to be attached via a linker. Nonetheless, surface-coupled TCO has been achieved by the silanization of oxide surfaces (silica, ITO) with a TCO–silane (available commercially), which can yield a TCO-terminated monolayer. As long as the TCO coverage is not so high as to cause TCO–TCO dimerization, the surface remains reactive to tetrazines [80]. A TCO–SAM on gold could be made by a thiol containing a TCO group, though one must be cautious that the TCO does not react with itself during assembly. In one demonstration relevant to biosensors, an antibody was site-selectively modified with a TCO via reaction with a unique cysteine on the Fc region, and a silicon electrode was functionalized with a tetrazine–silane; upon contacting the surface with the TCO–antibody, the antibody clicked onto the surface within minutes, yielding an oriented antibody layer [81]. This shows that either configuration (surface–tetrazine or surface–TCO) can work. The choice may depend on stability; tetrazines might need renewal, whereas TCO could be more stable on the surface if protected from light and on any additional functionality desired. Polymeric and nanostructured electrodes also benefit from IEDDA chemistry. For example, hydrogels used in biosensor designs, such as a gel that interfaces with skin and contains embedded electrodes, have been crosslinked using tetrazine–norbornene click reactions, which is a variant of IEDDA, where norbornene is less reactive than TCO but still undergoes IEDDA with tetrazines. This enables the formation of a biocompatible gel network in situ under very mild conditions, potentially around fragile electronics or biomolecules. A notable demonstration involved crosslinking a protein-loaded hydrogel atop an electrode using a tetrazine–TCO reaction, where the network formed almost instantaneously, trapping the protein in proximity to the electrode for sensing [82,83,84,85]. In carbon nanotube or graphene-based flexible electrodes, IEDDA can be a way to attach functional polymers or recognition elements without perturbing the conductive backbone. For instance, a tetrazine-functional polymer can wrap around a CNT and then be clicked to TCO-modified aptamers, creating a functional CNT biosensor in one step, whereas multi-step covalent modification of CNTs could disrupt their conductivity. IEDDA’s gentle nature (no catalysts, room temperature) is particularly suited for such nanomaterial hybrids.

The IEDDA reaction is essentially diffusion-controlled under typical conditions; unlike other click reactions, its performance is less sensitive to solvent and temperature, and more limited by how effectively the two reactants can encounter each other. Concentration and diffusion are thus key for ensuring a good supply of the solution-phase reagent to the surface by gentle stirring or convection will maximize the rate. Because the reaction is so fast, often, the rate of mixing or mass transport becomes the bottleneck once one component is in excess. In practice, simply shaking or agitating the sensor in the reagent solution is enough; there is no need for vigorous conditions. Temperature can affect it as rates roughly double with 10 °C increase, per typical Arrhenius behavior. However, since it is already fast at ambient temperatures, most conjugations are done at 20–25 °C. Notably, performing the reaction at 4 °C (for very sensitive proteins) is still feasible because IEDDA will proceed where slower SPAAC might stall at cold temperatures. IEDDA tolerates fully aqueous environments well, since tetrazines are often somewhat hydrophobic (many have aromatic substituents), but they can be formulated in aqueous buffer with a small percentage of ethanol or acetonitrile if needed. Some tetrazine reagents have polar sulfonate groups to increase water solubility for biological use [86].

Trans-cyclooctenes are usually hydrophobic hydrocarbons. However, when attached to proteins or hydrophilic linkers, they are effectively in a water-compatible form. Thus, one rarely needs organic solvents for TCO–tetrazine coupling on surfaces. An interesting condition aspect is pH, where tetrazine reactions do not require any particular pH (unlike, say, EDC coupling, which needs activation at a certain pH). As long as the pH is not extreme enough to degrade the tetrazine, which can hydrolyze in very basic conditions, the reaction will go smoothly. This flexibility allows IEDDA coupling to be carried out in the buffer that best preserves the biomolecule’s activity (e.g., pH 7 for antibodies or pH 5 for certain enzymes), thereby helping maintain functionality during immobilization. There are photostability considerations that must be made: if the functionalized surface is exposed to light, tetrazines may photo-bleach and/or undergo side reactions with light-generated radicals. For this reason, conjugations are commonly performed under subdued lighting or with appropriate protection, particularly when fluorescent tetrazines are employed. In practice, routine ambient laboratory lighting is generally acceptable; however, prolonged UV exposure remains a concern and should be avoided. More broadly, applying IEDDA click chemistry can confer highly desirable performance characteristics in wearable biosensors. Because immobilization is both rapid and gentle, bioreceptors retain high functionality: enzymes often retain substantial catalytic activity, and antibodies maintain strong antigen affinity. The short reaction time also enables tighter control over surface architecture. For instance, during the construction of multilayer assemblies, fast coupling allows each layer to be added in a controlled sequence without extended incubation periods that could otherwise permit intermediate layers to rearrange or degrade, supporting the fabrication of more reproducible multilayer interfaces. One of the most compelling implications of IEDDA for sensing is the potential for ultralow background, real-time readout strategies. Since tetrazines can quench fluorescence and become fluorescent upon ligation, some electrochemical platforms have explored dual-mode detection schemes. In these designs, an analyte-triggered tetrazine–TCO reaction at the electrode surface not only immobilizes an electroactive label but also switches on fluorescence as an orthogonal confirmation signal [87]. While this represents a hybrid approach, it illustrates how reaction design can be used to amplify detection. Even in purely electrochemical implementations, IEDDA-based immobilization typically yields highly stable surface attachments with minimal leaching.

A comparative assessment of surface-attachment strategies reported that a redox enzyme conjugated via the tetrazine–trans–cyclooctene (TCO) ligation retained its activity over substantially more operational cycles than an analogous enzyme immobilized by simple adsorption, highlighting the stability advantage of covalent IEDDA anchoring. An additional implication concerns response speed. In sensor formats, where the analyte-recognition event itself is coupled to a click reaction, the ultra-fast kinetics of the tetrazine–TCO pair could, in principle, translate into near-immediate signal generation upon analyte presence. Such concepts are actively explored for signal amplification. For example, by designing schemes in which each analyte-binding event initiates covalent capture of multiple reporter molecules through a cascade of click reactions. From the standpoint of wearability and potential in vivo interfacing, IEDDA is arguably the most amenable click reaction for integration with living systems. Notably, it has been applied in vivo for pre-targeted imaging in humans, e.g., using radiolabeled tetrazines to bind TCO-tagged antibodies in patients, supporting its feasibility, safety, and efficacy at that scale [88]. Translating this paradigm to wearable biosensing, one can envision future transdermal patch platforms in which one reactive partner resides in the body and the complementary partner is presented on the patch; their ligation would then generate a measurable signal directly on the device. The bioorthogonality and exceptional speed of IEDDA make such forward-looking concepts scientifically plausible. Thus, tetrazine–TCO click chemistry combines unprecedented reaction kinetics with strong biocompatibility, enabling high-efficiency functionalization of sensor interfaces and opening avenues for rapid, responsive biosensing, provided that the reactive handles are handled carefully to preserve their integrity and are deployed in a deliberate, well-controlled sequence.

### 2.4. Thiol-ene/Yne Photoclick Chemistry for Soft and Patterned Interfaces

The interfacial problem on soft wearable substrates is often different from that on flat noble metal electrodes: polymer films, hydrogels, and textiles frequently do not support the same SAM-based logic that works on gold. EDC/NHS coupling offers limited control over spatial patterning and mechanical compliance [89]. In this context, thiol-ene/yne photoclick chemistry is valuable because it uses light to define when and where covalent attachment occurs. Mechanistically, a thiyl radical, generated photochemically (typically in the presence of a photoinitiator), adds across a C=C or C≡C bond to form thioether- or vinyl sulfide-containing products. For wearable biosensors, the distinctive advantage of this chemistry is therefore not merely covalent bond formation, but the ability to localize surface modification, crosslink soft materials, and graft biomolecules under mild conditions on substrates that are difficult to address with classical SAM-based strategies. This feature set makes thiol-ene/yne particularly attractive for polymer-coated electrodes, hydrogels, elastomers, and textile interfaces. Vinyl- or alkyne-bearing primers can be introduced on Au SAMs, diazonium-modified carbons, silanized oxides, or polymer networks, after which thiol-containing probes can be immobilized in a spatially resolved manner. Conversely, biomolecules or coatings bearing terminal alkenes/alkynes can react with thiol-presenting surfaces or crosslinkers. Because the reaction is triggered only upon illumination, it enables micropatterning, localized attachment, and on-demand formation of antifouling or mechanically compliant networks directly on the device. Thiol–yne variants can also provide higher functional density or crosslink density because each alkyne can undergo sequential thiol additions. Norberg et al., for example, demonstrated aqueous thiol-ene/yne photocoupling of carbohydrate ligands to surfaces under mild conditions while preserving lectin-binding activity [90]. In practical terms, thiol-ene/yne chemistry is often most useful when the design problem is patterning or network formation rather than maximal reaction orthogonality.

The limitations are equally important. First, the chemistry depends on light delivery and radical generation, which can complicate scale-up and impose compatibility constraints for radical-sensitive biomolecules. UV-driven protocols may damage proteins, nucleic acids, or polymer substrates unless exposure is brief or shifted to longer wavelengths using visible-light photoinitiators. Second, oxygen can quench radical intermediates and reduce coupling efficiency, making illumination intensity, atmosphere, and photoinitiator choice important process variables. Third, if biomolecules carry multiple thiols, uncontrolled crosslinking or multilayer formation may occur unless the interface is designed around a single reactive thiol or low surface density. Finally, while thioether linkages are robust under normal sensing conditions, thiol-ene/yne is less attractive than triazole-based clicks when the fabrication workflow requires extremely oxidative cleaning or when catalyst-free bioorthogonality in highly complex biological media is the dominant priority. Accordingly, thiol-ene/yne photoclick chemistry should be viewed as a complementary interface-engineering strategy rather than a universal replacement for CuAAC, SPAAC, or IEDDA. Its strongest niche in wearable electrochemical biosensors lies in soft-material integration: photopatterned enzyme or aptamer domains, covalently functionalized hydrogel coatings, and polymer/textile substrates that benefit from localized, mechanically compliant surface chemistry. Because the preferred route for installing clickable handles is primarily substrate-dependent rather than unique to thiol-ene/yne chemistry, these material-specific activation strategies are compared later in Section 5 and summarized there as a substrate-selection framework (Table 4).

## 3. Building the Complete Interface: Orientation, Spacing, and Antifouling Design

It is worth noting that the tiny molecular bridges connecting the bioreceptors to the wearable biosensor surface are not just for show; they actually influence the ease of electron flow and the biointerface. An important trade-off is the stiffness versus flexibility of these molecular links, as this influences the distance of the electrode from the redox-active biomolecule and the efficiency of electron tunneling. For rigid linker molecules, consisting of π-conjugated groups, the bioreceptor is held rigidly, and the electron tunneling is accelerated by the availability of a “delocalized path.” Indeed, the conductivity of self-assembled monolayers (SAMs) increases significantly by increasing the density of conjugated π-bonds in the linker [91,92] or oligophenylene ethynylene-based SAMs; here, the electron tunneling attenuation factors (β) are lower than for flexible alkane thiols, indicating a greater efficiency of the interface for electron tunneling. On the other hand, the flexibility of the linker—long alkyl chains and flexible poly(ethers) such as poly(ethylene glycol)—gives the system greater scope to move and “drift apart,” slowing down the electron tunneling. In the case of enzyme-based biosensors, the bioelectrocatalytic current is very sensitive to the folding of the flexible linker and its length; shortening the flexible linker dramatically increases the current by shortening the distance of the electron tunneling. In particular, for multi-domain redox enzymes, the binding of Ca^2+^ to a flexible linker and shortening the distance of the inter-domain linker increases the efficiency of direct electron transfer (DET). This is consistent with the classic distance threshold of the electron tunneling distance established by Dutton’s rule, where the distance has to be <14 Å for efficient eT to occur [93]. In general, therefore, rigid and short linker molecules are desirable for efficient electron tunneling and thus efficient biosensor signals and low detection limits. However, it is important to be careful: overly rigid and short linker molecules can actually misalign the bioreceptor and decrease its activity. A balanced approach is often needed, integrating semi-flexible segments that allow the biomolecule to orient optimally without excessive distance that would impede electron flow.

**Figure 4:**
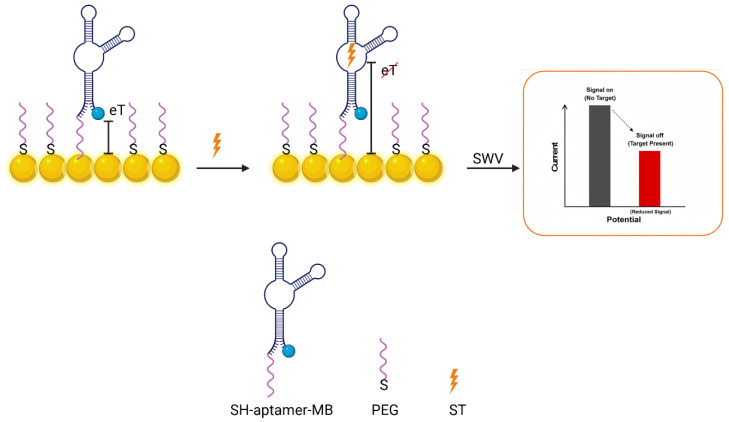
Schematic representation of the electrochemical aptasensor assembly for serotonin detection utilizing a PEG backfilling strategy [94].

Beyond electron transfer considerations, linkers serve as molecular spacers and antifouling moieties that modulate surface chemistry and stability. In wearable biosensors, the nonspecific adsorption from complex samples can severely degrade the signal-to-noise ratio. Hence, antifouling linkers are commonly employed. Poly(ethylene glycol) (PEG) is a gold standard antifouling polymer that can be incorporated into linkers to resist protein adsorption [94]. For instance, thiolated PEG chains terminating in a click-reactive group (azide or alkyne) can form a SAM on gold that presents a hydrophilic, protein-repelling interface while still allowing specific bioreceptor attachment via click chemistry [95]. Such PEGylated linkers dramatically reduce background noise and improve biosensor selectivity in complex media. In one recent design, a PEG-functionalized aptamer interface showed minimal nonspecific binding in human serum, leading to enhanced antifouling capability and preservation of sensitivity toward serotonin (ST) [94] (Figure 4).

**Figure 5:**
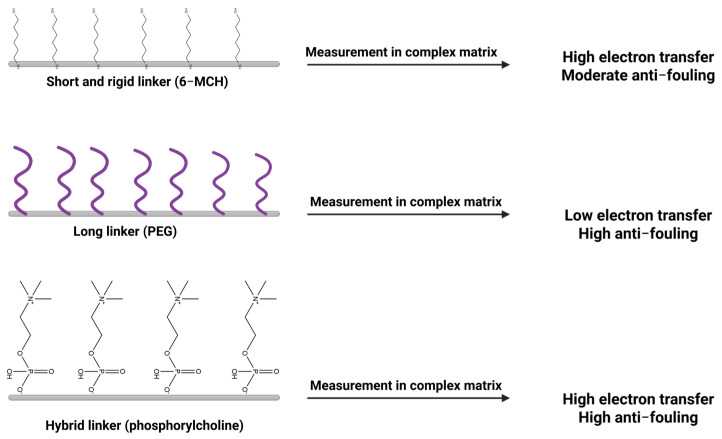
The linker choice affects the electron transfer rate and antifouling feature. The shorter the linker, the higher the electron transfer rate with low–moderate antifouling. The longer the linker, the lower the electron transfer rate with high antifouling. The hybrid or specific linkers provide both a high electron transfer rate and antifouling.

Integrating antifouling segments into linkers often imposes a trade-off between surface passivation and electron transport, but click chemistry offers ways to mitigate this. Conductive linkers can be designed as hybrids, for example, a conjugated aromatic backbone for electronic conduction coupled with terminal PEG brushes for antifouling. The click-coupled triazole itself is a rigid, conjugated unit that can participate in electron conduction. Moreover, click reactions enable attaching redox-active spacers (like ferrocene or quinone groups) between the electrode anchor and the bioreceptor [96], effectively wiring the biomolecule for direct electron transfer. By careful linker molecular engineering, one can tune the electron-transfer kinetics while also conferring stability and reproducibility to the surface. Moreover, the covalent triazole link formed by azide–alkyne cycloaddition is chemically stable and withstands mechanical stress or solvent exposure, which is a vital attribute for wearable sensors operating in vivo or in sweat. Thus, rational linker design, balancing rigidity for fast electron tunneling with flexibility for bioreceptor functionality, and incorporating antifouling/hydrophilic elements, is crucial for optimizing biosensor performance. These design choices directly impact the sensor’s signal strength (via eT rate), background noise (via fouling resistance), and operational stability (via strong covalent anchoring and preserved biomolecule activity) [97,98]. Taken together, the comparison in Table 2 suggests a practical design rule rather than a simple menu of linker classes. If the sensing mechanism is direct-electron-transfer-limited or relies on a surface-confined redox reporter, the first priority should be a short, rigid, or partially conjugated tether, while antifouling elements should be introduced only as minority diluents or distal segments. By contrast, if long-term operation in sweat, serum, or interstitial fluid is the dominant challenge, PEG/OEG or zwitterionic spacers may justify some loss in electron-transfer rate because they suppress nonspecific adsorption and baseline drift. In wearable devices, hybrid architectures are therefore often optimal: a compact conductive junction for signal transduction combined with localized antifouling components that protect the interface without converting the entire sensing layer into an insulating film. Table 2 should be read in that decision-oriented way, rather than as a catalog of linker options. Figure 5 shows the trade-off between electron transfer rate and antifouling according to the linker choice.

### 3.1. Controlling Bioreceptor Orientation

Maintaining a favorable and uniform orientation of immobilized bioreceptors is essential for high sensor activity, especially in enzymatic and immunosensing interfaces. Random attachment of enzymes or antibodies onto surfaces can lead to a significant fraction of the binding or catalytic sites being sterically blocked or facing the electrode, rendering them inaccessible to the target analyte [102]. This not only diminishes the signal by lowering the effective surface coverage of active receptors but also introduces variability and poor reproducibility. Therefore, site-specific immobilization strategies are employed to orient bioreceptors in an upright and functional manner. Click chemistry has emerged as a powerful tool to achieve it by selectively attaching biomolecules through a predefined site without perturbing their active domains [103,104]. The general approach is to introduce a unique bioorthogonal handle (azide or alkyne) at a specific location on the bioreceptor, often at a terminus or a region that will orient the molecule favorably, and then to covalently link it to a complementary-functionalized surface via CuAAC or strain-promoted cycloaddition. This covalent site-targeted coupling yields a uniform monolayer of biomolecules with their recognition sites exposed, and the resultant triazole linkage is formed in high yield under mild conditions, preserving biomolecular activity. The outcome is a sensor surface with enhanced activity and stability: for example, azide–alkyne click attachment of aptamers on graphene produced significantly improved biosensing performance, owing to the well-defined orientation and density of probes on the surface.

Orientation is determined not only by the attachment site, but also by the geometry of the tether connecting the bioreceptor to the surface. Very short and rigid linkers can improve electronic coupling and reduce tunneling distance, yet they may force antibodies, enzymes, or aptamers into sterically unfavorable poses that partially mask the recognition domain. Conversely, highly flexible linkers such as long alkyl chains or PEG segments often preserve conformational freedom but can increase the average separation between the electrode and redox-active center, thereby attenuating electron transfer. The practical design goal is therefore a balanced tether: short enough to minimize electron-transfer penalties, but sufficiently compliant to allow the biomolecule to adopt an active, outward-facing configuration. In this sense, orientation control and linker geometry must be engineered together rather than treated as separate design variables.

**Figure 6:**
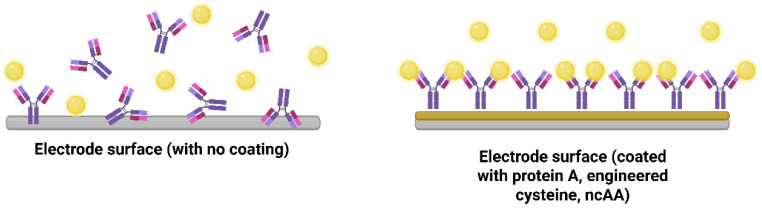
The biorecognition element immobilization efficiency dramatically increases when the proper coating is engineered onto the electrode surface.

Oriented attachment via click chemistry has been shown to increase biosensor sensitivity by orders of magnitude compared to random immobilization [105]. For instance, Trilling et al. compared a single-site azide-labeled V_H_H antibody to a randomly multi-azido labeled one on a cyclooctyne-functionalized surface, where the site-specific oriented probe achieved an ~800-fold higher detection signal [103]. This striking improvement stems from preserving the proper upright alignment of the antigen-binding domains in the oriented case, whereas randomly attached antibodies suffer from many inactive or misoriented units. Importantly, the oriented monolayer is also more reproducible (each molecule adopts a similar configuration) and often more stable. The covalent, site-specific anchoring avoids the weak links and conformational strain that can occur in random chemisorption, thus reducing biomolecule desorption or denaturation over time. To achieve such controlled orientation, several bioconjugation techniques can be combined with click chemistry. Site-directed mutagenesis is a common approach for proteins, in which a unique functional group can be genetically introduced at a desired position away from the active site. For example, an exposed cysteine can be engineered at the tail of an enzyme or antibody, which is subsequently modified with an alkyne maleimide, allowing the protein to click onto an azide-functionalized surface exclusively through that cysteine site. Similarly, noncanonical amino acids bearing click handles, such as p-azido-L-phenylalanine or azidohomoalanine, can be incorporated at specific residues during recombinant expression. Shen et al. demonstrated this by installing azide groups on an IgG antibody and clicking it onto alkyne-modified gold nanoparticles, achieving oriented attachment via CuAAC [105]. A caveat is that protein engineering may slightly affect folding or activity if the mutation site is suboptimal, where careful selection of labeling sites (at an IgG Fc terminus or a flexible enzyme loop) is therefore crucial. Another powerful method is glycoengineering of antibodies, in which IgG antibodies are modified to possess N-linked glycans on their Fc region. They can be chemically or enzymatically modified with click functional groups without altering the antigen-binding Fab arms. One strategy is to oxidize Fc glycan sugars to aldehydes and attach an aminooxy–PEG–alkyne or directly incorporate azido–sugars (azido–fucose or azido–sialic acid analogs) into the Fc glycan [105]. The antibody can then be immobilized via a click reaction (SPAAC with a surface-cyclooctyne), effectively anchoring it through the Fc glycan. This ensures the Fab regions are oriented outwards, maximizing binding while covalently locking the antibody on the surface. Because the Fc glycan site is distant from the antigen-binding interface, such conjugation preserves immunoreactivity. Site-specific bioconjugation tags can also be used (an LPETG tag on a protein for sortase-mediated attachment of an azide). However, it must be noted that the click chemistry’s advantage is its direct and bioorthogonal coupling. By controlling orientation, higher effective surface coverage of active bioreceptors and more uniform binding kinetics can be achieved, which translates to improved sensor performance. Signals are stronger with a higher signal-to-noise because each immobilized biomolecule contributes fully to target recognition or catalysis. Specificity is enhanced since nonspecifically adsorbed or denatured proteins are minimized. Additionally, oriented layers tend to be more stable over time [106,107]. For example, an antibody properly oriented via Fc-binding or click attachment retains activity over weeks, whereas a randomly attached one may lose activity as misoriented parts gradually denature or detach [102]. The combination of click chemistry with protein engineering thus offers a robust route to create smart surfaces where bioreceptors are not only firmly attached, but also optimally presented. This directly impacts sensor reproducibility, since lot-to-lot variation decreases when each assembly yields the same ordered architecture. In multiplexed wearable devices with many sensing sites, such orientation control is key to ensuring each site responds consistently. Overall, site-specific click immobilization of bioreceptors markedly boosts sensitivity, lowers detection limits, and improves the reliability of wearable biosensors by marrying molecular precision with surface chemistry [102]. The strategies summarized in Table 3 are not equivalent from an electrochemical design standpoint. Fc-binding affinity layers offer the simplest route to upright antibody presentation because they avoid direct antibody derivatization, but the added Protein A/G/L layer increases the separation between the electrode and the recognition element and can introduce a noncovalent weak point; they are therefore attractive for label-based immunosensors but less ideal for interfaces that depend on short electron-transfer pathways or extreme shear stability. Fc glycan-directed coupling reduces this problem by creating a distal, more uniform single-point attachment without introducing as much interfacial bulk, although it requires extra chemoenzymatic processing. Engineered single cysteines and noncanonical-amino-acid-installed click handles provide the highest geometric precision and usually the shortest fully covalent path to the surface, making them the strongest options when direct electron transfer, redox–reporter coupling, or strict reproducibility is the priority. By contrast, streptavidin/biotin, DNA-directed immobilization, and tag–catcher systems excel in modularity and programmability, but they introduce additional macromolecular layers or flexible spacers, which can be beneficial for accessibility yet detrimental when interfacial compactness is critical. Thus, the preferred orientation strategy depends not only on biological activity, but also on whether the sensor is electron-transfer-limited, mechanically stressed, or intended for low-cost routine fabrication. Figure 6 illustrates the efficiency of biorecognition element immobilization onto the electrode surfaces with and without coating.

### 3.2. Mixed Monolayers, Spacers, and Antifouling Architecture

Even with well-designed linkers and oriented coupling, the density and distribution of immobilized species on the surface must be carefully controlled to prevent crowding and interference. Immobilizing a high concentration of functional groups on a surface can lead to steric hindrance, inhomogeneous coverage, and unintended perturbation of electron transfer pathways [111]. The solution is to form mixed self-assembled monolayers (mixed SAMs) composed of both functionalized and diluent molecules. In a mixed SAM, one component carries the click-reactive group for bioreceptor attachment, while the co-component is an inert “spacer” molecule that dilutes the surface density of the reactive sites. This strategy is widely regarded as the optimal way to regulate biomolecule arrangement on surfaces [112]. By adjusting the ratio of functional thiol to diluent thiol during assembly, the average spacing between neighboring bioreceptors after immobilization can be tuned. Proper spacing is critical, since it ensures that large biomolecules (antibodies, enzymes) do not sterically block or denature each other when attached. It also leaves enough of an interstitial area for target analytes or redox mediators to access the interface [113,114]. Surface crowding is known to impede sensor performance, where densely packed DNA or aptamer probes can hinder target binding and slow hybridization kinetics, and closely spaced antibodies can exhibit reduced antigen binding due to steric clashes. Mixed monolayers alleviate these issues by creating a more homogeneous, less crowded landscape. Frederix et al. demonstrated that SAMs composed of a mixture of carboxyl-terminated thiols (for attaching antibodies) and hydroxyl-terminated or PEG-terminated thiols (as diluents) yielded superior biosensor interfaces compared to single-component films. The mixed SAM showed higher antibody loading in an active orientation, greater antigen binding capacity, and dramatically lower nonspecific adsorption of proteins, leading to enhanced sensitivity, stability, and selectivity [95]. In essence, the inert diluent molecules (e.g., oligo(ethylene glycol) units or short alkanols) act as molecular crowd control, spacing out the binding sites and presenting a protein-resistant background between them. Mixed monolayers are also the natural platform for integrating antifouling spacer chemistry into the interface. OEG/PEG backfillers hydrate the surface and suppress nonspecific adsorption, while zwitterionic diluents or peptide-based mixed-charge spacers can create even more strongly water-shielded interfaces in sweat, serum, or interstitial fluid. The trade-off is that highly hydrated and flexible spacers can increase the effective tunneling distance and reduce faradaic response if they dominate the sensing layer. Consequently, the most effective wearable interfaces usually use these components as minority diluents or post-immobilization backfillers around the clicked bioreceptor rather than as indiscriminately thick coatings. This architecture preserves target accessibility and antifouling performance while retaining a sufficiently short and conductive pathway between the recognition element and the electrode. Representative examples include PEG-backfilled serotonin aptamer interfaces and zwitterionic peptide-functionalized click biointerfaces, both of which reduce fouling without sacrificing analytical performance.

Zwitterionic linkers, containing equal positive and negative charges, are another powerful strategy. These linkers, based on phosphorylcholine or carboxybetaine groups, form highly water-shielded surfaces that prevent biofouling. They can be synthesized with terminal azides/alkynes for click attachment. In a specific example, a zwitterionic peptide was click-conjugated to an aptamer probe, yielding an ultralow-fouling biointerface that enabled biomarker detection in complex biological fluids [115,116]. Such mixed-charge peptides or polymers endow the sensor with superior resistance to fouling while providing functional handles for bioreceptor immobilization via CuAAC or SPAAC. Forming a mixed monolayer for a wearable biosensor might involve co-adsorbing a thiolated alkyne (for click coupling) with a thiolated PEG or alkane that has no reactive end. Only a fraction of surface sites (10–30%) carry the clickable group, while the rest are occupied by the diluent. This ensures that when bioreceptors are attached via click reaction, they are separated by gaps of an inert monolayer, avoiding clustering. These diluent spacers also help create a well-ordered film. On flat gold surfaces, alkanethiol SAMs naturally form close-packed structures. However, on nanostructured or rough electrodes often used in wearables, packing can be imperfect. Backfilling any defects or unoccupied sites with small hydrophilic thiols after the initial assembly is a common step to achieve monolayer uniformity. Such backfilling essentially produces a mixed SAM post hoc, plugging any gaps that could become hotspots for nonspecific adsorption. It also prevents lateral electron leakage through bare patches of the electrode [117]. The incomplete SAM coverage allows solution ions to directly access the electrode, which flattens impedance spectra and undermines reproducibility. By contrast, a densely packed mixed monolayer presents a consistent barrier with well-defined molecular pathways, yielding more reproducible charge-transfer resistance and capacitance readings [102]. Thus, the use of mixed monolayers leads to greater electrode passivation consistency and batch-to-batch reproducibility in sensor fabrication. When dozens of sensors must operate under one calibration (as in multiplexed wearable devices), such uniformity is indispensable.

**Figure 7:**
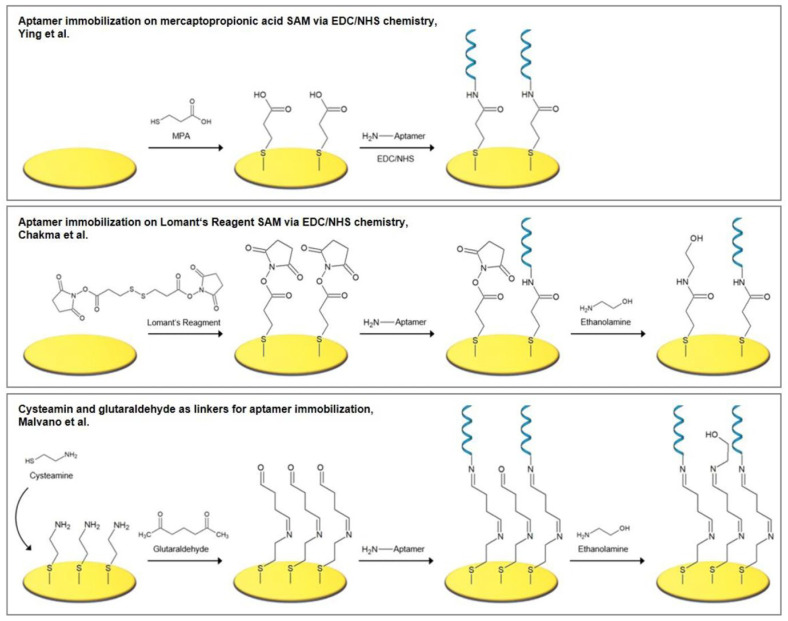
Schematic representation of the post-immobilization backfilling strategy using 6-mercaptohexanol (MCH). The MCH molecules displace nonspecifically adsorbed aptamers and form a mixed self-assembled monolayer (SAM), promoting an upright orientation of the DNA probes to enhance target accessibility and reduce nonspecific surface fouling [119].

Another benefit of mixed SAM design is the mitigation of electronic and steric disruption caused by the bioreceptors themselves. A fully dense monolayer of enzymes on an electrode, for example, can create a thick insulating layer that slows electron transfer to underlying redox reporters or electrode surfaces. Introducing diluents of shorter length can maintain nano-scale channels or a thinner effective film for electron tunneling [118]. In EIS-based biosensors, an optimally spaced monolayer strikes a balance between being tight enough to block nonspecific adsorption, but open enough to allow target binding and efficient Faradaic reactions [102]. Mixed SAMs are also frequently employed with binary functionalities, such as a mixture of a clickable group and an anti-fouling group. For instance, a gold electrode may be modified with a binary SAM of 11-mercaptoundecanoic acid for EDC/NHS coupling or click conversion to an azide and 6-mercaptohexanol. The mercaptohexanol not only dilutes the surface carboxyl/azide density but also confers some resistance to protein fouling and helps orient tethered biomolecules upright by preventing them from lying flat on the surface. In aptamer-based wearable sensors, this approach is standard, since the thiolated aptamer is co-assembled or post-assembled with mercaptohexanol, which greatly improves the DNA probe’s accessibility and reduces background currents [119] (Figure 7). Without such spacing, aptamers can form surface-induced secondary structures or multilayered aggregates, hampering target binding; with proper spacing, they remain flexible and functional. The net effect on performance is significant, with higher signal upon target binding, faster response due to less diffusional hindrance, and minimal drift due to fouling or desorption. Furthermore, mixed monolayers lend themselves to tunability.

**Figure 8:**
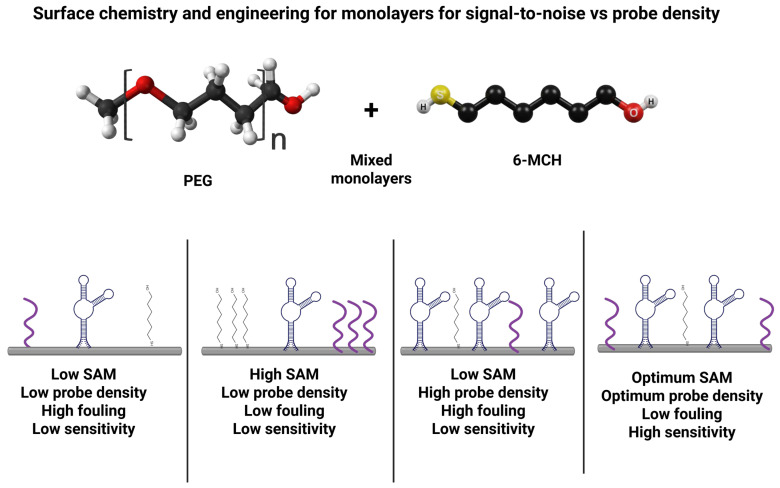
The relationship between SAM and probe density affects the fouling and sensitivity of the biosensor platform.

By varying the diluent proportion, the surface probe density can be modulated to an optimal level rather than an all-or-nothing coverage. This optimization can maximize the signal-to-noise ratio: too few probes give a low signal, but too many give high noise or low per-probe activity. An optimal intermediate coverage often exists, and mixed SAM chemistry provides a facile way to achieve it by simply changing solution composition during assembly [120]. Therefore, the design of mixed monolayers is a cornerstone of surface molecular engineering in wearable biosensors. It ensures that clickable functional sites are deployed in a spatially organized and non-interfering manner. By preventing overpacking, mixed SAMs preserve the biological activity of immobilized receptors and maintain efficient electron transfer pathways to the transducer [121]. They also impart robust antifouling characteristics when hydrophilic spacers are used, improving the long-term stability of sensors in protein-rich biofluids. It is equally important to note that mixed monolayers improve device reliability and reproducibility, since each sensor surface achieves a similar functional group spacing and coverage, which translates to consistent sensor responses across different devices and over time. From a translational perspective, this means calibration models remain valid, and sensor fabrication yields are high. Thus, through mixed-monolayer architecture, one can finely tune the interfacial chemistry to attain high signal, low noise, and durable performance, all of which are critical for real-world wearable biosensing applications. Figure 8 illustrates the mixed SAMs and probe density effects on the fouling and sensitivity of the biosensor systems.

## 4. Impact on Electrochemical Performance Metrics Under Wearable Deformation and Hydration

The performance metrics discussed below are direct readouts of how successfully an interface has moved beyond classical immobilization. In wearable electrochemical biosensors, however, these metrics cannot be interpreted as if the interface were static. The relevant question is not only whether a clicked bioreceptor is covalently attached on day 0, but whether the full linker–substrate architecture maintains a predictable electron-transfer pathway and a stable local microenvironment during bending, stretching, sweat exposure, and hydration–dehydration cycling. Accordingly, stability, sensitivity, and reproducibility are treated here in a wearable-specific sense, as consequences of how molecular junctions behave under dynamic deformation rather than under ideal undeformed conditions alone (i.e., bioconjugation chemistry). Therefore, it provides a direct bridge between molecular design and electrochemical sensor performance in a wearable-specific context, not merely a benchtop context. In this setting, the relevant electrochemical parameter is not a single static keT value measured on a pristine flat interface, but the distribution of electron-transfer pathways that remain accessible as the interface deforms, swells, rehydrates, and relaxes during use. Replacing conventional random coupling routes with site-specific click ligations enable a more uniform and predictable biointerface, which in turn improves key performance metrics including interfacial electron-transfer kinetics, operational stability, sensitivity, and assay-to-assay reproducibility. In particular, click-enabled, orientation-controlled immobilization can shorten and homogenize the effective electron-tunneling distance between the electrode and the redox-active center (or recognition layer), thereby engineering the dominant electron-transfer pathway at the interface [122]. The introduction of defined linkages such as 1,2,3-triazoles (from CuAAC) or thioether bonds (from thiol-ene photoclick) in the tether can modulate the heterogeneous electron transfer rate constant by altering the distance, rigidity, and conjugation of the molecular bridge. Marcus theory for electron transfer predicts an exponential decay of tunneling rate with distance and a strong dependence on electronic coupling. In other words, each additional ≈0.5–1 nm of an insulating linker can dramatically slow electron transfer kinetics (often an order-of-magnitude decrease per few Å of separation) due to the exponential tunneling barrier. Conversely, incorporating conjugated or rigid segments in the linker can enhance electronic coupling and reduce reorganization energy, thus partially mitigating the distance penalty. For example, Collman et al. covalently attached redox probes to gold via CuAAC and demonstrated the values spanning more than 60,000 s^−1^ and down to 1 s^−1^ simply by varying the bridge length and conjugation. Notably, the aromatic triazole ring formed by click coupling provided electronic coupling for tunneling similar to an ester linkage, indicating it does not severely insulate the electron pathway. In contrast, a flexible aliphatic thioether linkage (C–S–C) behaves more like a saturated alkane, which tends to have a larger tunneling decay constant (β), thus slowing electron transfer when elongated [22]. From a theoretical standpoint, the Butler–Volmer formalism connects a lower electron transfer kinetics to a higher activation overpotential. This means that, if a click linkage were overly insulating, the larger peak separations in cyclic voltammetry (CV) and increased charge-transfer resistance in impedance spectra would be observed. Mathematically, electrochemical impedance spectroscopy (EIS) confirms that adding long or non-conjugated tethers raises the interfacial charge-transfer resistance, consistent with slower electron kinetics. Meanwhile, CV measurements of click-functionalized monolayers often show near-ideal Nernstian behavior (small peak separations) when the linkers are short and conjugated but deviate toward quasi-reversible kinetics as the tunneling distance grows. These effects align with Marcus–Gerischer models, where electron tunneling rate constants decay exponentially with distance and are modulated by the linker’s electronic structure. Scanning electrochemical microscopy (SECM) studies further support this, as demonstrated by early work with alkanethiol SAMs, which showed drastically reduced eT rates through long alkane chains [22]. By replacing part of the chain with a rigid aromatic triazole via click coupling, the tunneling efficiency can be enhanced. Hence, click-derived linkers allow engineering of the electron transfer rate. Specifically, a short, π-conjugated click linkage can facilitate faster electron transfer as higher currents and more reversible voltammetry, whereas an overly long or flexible linkage will attenuate tunneling and require larger driving potentials to achieve the same current [123].

On the experimental front, numerous studies have quantified these phenomena. Devaraj et al. assembled mixed monolayers on Au with varying fractions of azide-terminated thiols and clicked different-length acetylenes to them, obtaining a tunable series of electron-transfer rates [22]. They observed that triazole-linked redox centers could still exchange electrons rapidly when the spacer was short and conjugated, but the electron transfer rate dropped as additional methylene units or rotatable bonds were inserted (consistent with an exponential distance dependence). Such findings reveal that the linker design in terms of length and rigidity in click-immobilized biosensors is not merely a structural detail but a critical determinant of electron transfer kinetics. Fortunately, the chemoselectivity of click reactions enables precise linker architectures. By using a cyclooctyne with an extended conjugation for SPAAC, it can yield a rigid, shorter path compared to a long EDC/NHS-derived amide polymer. Ultimately, by applying Marcus–Butler–Volmer principles, researchers can deliberately tune click linker structures to optimize electron transfer rate for rapid electron delivery, which is a crucial factor in achieving fast response times and low overpotentials in wearable electrochemical sensors [124,125].

### 4.1. Electron Transfer and Signal Drift at Dynamically Deforming Interfaces

Click-enabled, orientation-controlled immobilization can shorten and homogenize the effective electron-tunneling distance between the electrode and the redox-active center (or recognition layer), thereby engineering the dominant electron-transfer pathway at the interface [126,127]. Marcus-type models still apply the tunneling rate decays exponentially with distance and depend strongly on electronic coupling, so each additional insulating segment can substantially attenuate kET. Conversely, rigid or partially conjugated junctions can preserve electronic coupling and reduce the distance penalty [128]. For this reason, compact triazole-containing linkages are often more favorable for interfacial electron transfer than long saturated or highly mobile tethers when other structural variables are comparable. For wearable devices, however, kET should be viewed as a mechanically and hydration-modulated parameter rather than a fixed value measured on an undeformed laboratory electrode. A compact triazole-rich junction can be advantageous because it limits conformational wandering and helps preserve a short donor–acceptor separation during bending [129]. However, this is not a universal rule: if the neighboring primer layer, SAM, or substrate anchor is brittle, a highly rigid junction can transmit strain more directly to the weakest adjacent element and thereby accelerate local cracking, delamination, or loss of interfacial order. By contrast, flexible PEG- or OEG-rich spacers can dissipate strain and improve antifouling performance, but under tensile deformation and repeated hydration, they can extend, reorient, or swell, broadening the distribution of donor–acceptor distances and therefore broadening the distribution of instantaneous electron-transfer rates [130]. In practice, this often appears not as catastrophic bond failure, but as lower faradaic current, increased charge-transfer resistance, broader peak separation, or slow baseline drift during long wear.

The same logic explains why reagent identity matters beyond click yield alone. In SPAAC-derived interfaces, bulky cyclooctynes such as DBCO can perturb soft or highly hydrated interphases more strongly than smaller or more hydrophilic handles [131]. Their aromatic and relatively hydrophobic character can alter local wetting and, in swollen polymer or PEG-rich films, accentuate interfacial reorganization under continuous sweat exposure. This point is consistent with earlier observations in this review: hydrophilic PEG-linked azide coatings may swell and partially delaminate when exposed to bulky DBCO reagents, even though the resulting triazole bond itself remains chemically robust. Thus, in wearables, electrochemical drift may arise; this is not because the click adduct breaks, but is because hydration and deformation continuously change local film thickness, ion accessibility, dielectric environment, and effective tunneling distance. The most effective wearable architecture. Therefore, it separates two design functions rather than forcing a single linker to do everything. The electron-transfer pathway should remain compact and structurally defined near the transducer, whereas compliance and antifouling are better introduced through mixed monolayers, compliant primer layers, polymer networks, or distal PEG/zwitterionic segments that do not dominate the entire tunneling path. In this sense, the decisive design variable is not simply “rigid versus flexible,” but where rigidity is placed, where compliance is placed, and whether the substrate, primer layer, and click junction deform cooperatively.

### 4.2. Stability and Shelf-Life

One of the most well-known advantages of click-immobilized bioreceptors is the robustness of the resulting linkage under operational and storage conditions. However, the operational shelf-life of the complete biointerface is controlled not only by the intrinsic stability of the click-derived bioreceptor linkage but also by the robustness of the linker–substrate junction that supports the entire recognition layer. Specifically, it stands out when compared to classical carbodiimide chemistry. Click reactions form bonds (C–C, C–N, or C–S) that are essentially inert to hydrolysis and resistant to bond cleavage under physiological conditions. For example, the 1,2,3-triazole ring formed by azide–alkyne cycloaddition is a thermodynamically stable heterocycle that does not hydrolyze or revert, and thioether bonds (from thiol-ene reactions) are likewise resistant to oxidation or hydrolysis in aqueous environments. In contrast, the amide bonds obtained via EDC/NHS coupling, while covalent, can be points of vulnerability. On the other hand, amide linkages can slowly hydrolyze under extreme pH or elevated temperatures, and more critically, the EDC/NHS process often yields some unstable intermediates and side products on the surface. The *O*-acylisourea active ester formed during EDC activation is notorious for its short half-life in water, leading to incomplete coupling and residual surface carboxylates that can later hydrolyze or cause nonspecific adsorption [132]. Even after successful amide formation, proteins attached by multi-point EDC chemistry may detach over time if any single anchoring bond is broken, since random multi-lysine attachments do not guarantee a strong single-point hold. By using bioorthogonal click linkages, the sensor interfaces exhibit far superior long-term stability. For instance, surfaces functionalized with azide groups can be prepared and stored dry for months without losing reactivity. Simonian et al. report that azido SAM chips remain stable for at least several months at 4 °C, whereas EDC-activated surfaces must be used immediately due to rapid hydrolytic degradation [133]. The completed click-coupled bonds themselves (triazoles, etc.) also endure harsh conditions. Sensors built with CuAAC-linked enzymes have shown minimal loss of activity after weeks of continuous use in aqueous media, attributable to the hydrolytic resistance of the triazole connection. In one comparison, a peptide-functionalized hydrogel crosslinked via a thiol-yne click retained the peptide’s bioactivity for cell signaling significantly better than an analogous EDC-crosslinked hydrogel, where the EDC method had inactivated a portion of the peptide (losing its biological effect) [134]. This illuminates that click methods often preserve biomolecule function due to gentler reaction conditions and avoidance of excessive functional group modifications, where EDC can create unwanted intra- or inter-molecular crosslinks that denature proteins.

For wearable biosensors, stability must be maintained under real-world stressors, including temperature swings, humidity/sweat exposure, and mechanical strain. In this case, the rigidity and strength of click linkages shine, since a recent study on a wearable sweat sensor employing a reticular click-crosslinked hydrogel interface demonstrated stable performance over repeated daily use. It exhibited a negligible signal drift in high-humidity, saline sweat conditions, where classical interfaces often degrade. Similarly, click-functionalized enzyme electrodes have been reported to retain activity after weeks of storage at room temperature or refrigeration, with little–no loss in sensitivity, whereas analogous EDC/NHS-prepared electrodes showed significant signal decline over the same period. The reason is that the covalent anchoring via click is exceptionally robust, since there are no labile ester linkages or unreacted groups left to cause detachment or hydrolysis. In one example, a CuAAC-linked monolayer of a redox enzyme on gold maintained over 90% of its initial current response after 14 days in a pH 7.4 buffer at 37 °C, far outperforming a randomly adsorbed enzyme layer, which lost over half its response. Even under aggressive chemical conditions, click-assembled interfaces hold up. The covalently grafted GNR network provided a stable, conjugated interface that did not delaminate or degrade, ensuring consistent sensor function. Moreover, the orthogonality of click reactions means that a surface in one step, without harsh catalysts or byproducts that could attack the biomolecule or substrate, can be functionalized. For instance, strain-promoted azide–alkyne cycloaddition (SPAAC) can tether antibodies to a flexible textile electrode in a gentle buffer, avoiding the low-pH and carbodiimide reagents of EDC that often denature proteins. In the end, the use of click chemistry in wearable biosensors yields interfaces that are molecularly locked in place, offering superior shelf-life, stable for months of storage and operational durability, resisting hydrolysis and sustaining bioactivity in heat, humidity, and physiological fluids. These attributes are critical for devices intended for continuous monitoring, where frequent recalibration or replacement due to interface degradation is undesirable.

### 4.3. Sensitivity and Limit of Detection (LOD)

The sensitivity of an electrochemical biosensor, often defined by its signal-to-noise ratio for low analyte levels and the achievable limit of detection, is strongly influenced by the structure and coverage of the biorecognition layer. Click chemistry has enabled surface architectures with high probe density and optimized orientation, which in turn amplify the analytical signal. A key advantage of click-based bioconjugation is the near-quantitative coupling efficiency and site-directed immobilization. Because click reactions can approach 100% yield of attachment (limited only by sterics), a very high packing density of active receptors is achieved on the sensor surface. In contrast, classical EDC/NHS often achieves incomplete and random coupling, leaving fewer active bioreceptors and many dead sites. Maximizing surface coverage with properly oriented bioreceptors increases the fraction of the electrode area that actively participates in analyte recognition or catalysis, thereby boosting the current or potential change per analyte concentration. Indeed, by controlling the ratio of azide-terminated thiols in a mixed monolayer, the surface coverage of clicked biomolecules with precision can be tuned, and every available azide can be converted to an attached probe [67]. This quantitative loading means the signal can be scaled up predictably without the trial-and-error often needed for random adsorption processes. Equally important is the orientation of the immobilized biomolecules. In this context, click chemistry allows one to tether biomolecules through specific sites (a unique azido-handle on an engineered antibody or enzyme), ensuring that all the recognition elements are uniformly oriented with their active sites exposed. This oriented immobilization drastically improves the effective binding or catalytic capacity per molecule. A recent study by Kolanovic et al. illustrates this effect. They site-specifically attached a lectin protein on electrodes via bioorthogonal click ligation of a genetically encoded azide and observed a ~12-fold increase in binding sensitivity for its target glycoprotein compared to a randomly immobilized lectin via conventional NHS coupling [19]. The click-oriented lectin had all its binding sites facing the solution, whereas random attachment led to many molecules improperly oriented or partially inactive, thus yielding much lower signals. This example demonstrates how signal-to-noise ratios improve when every immobilized receptor is optimally positioned, where the specific analyte signal is higher, and background noise from non-functional or denatured receptors is lower. Likewise, in immunosensors, site-directed click attachment of antibodies (through a cyclooctyne at the Fc region) yields an upright antibody configuration. Trilling et al. showed that uniformly oriented capture antibodies can dramatically enhance sensitivity relative to a mix of orientations [103], which is in line with observations in many click-functionalization studies that proper orientation boosts target accessibility and electron transfer efficiency to reporting redox probes.

Another way click chemistry strategies improve sensitivity is by enabling dense yet organized monolayers that maximize analyte capture without steric hindrance. Traditional methods often face a trade-off, as a high grafting density can lead to steric crowding and diffusional barriers that actually reduce sensor response. However, with click-assembled multilayers or polymeric linkers, spacer units (PEG chains, or use of a multivalent click tree) can be incorporated that keep biomolecules sufficiently separated even at high surface density. The result is high loading of active biomolecule per unit area, each still accessible to the analyte. Empirical examples abound where click-based immobilization yields orders-of-magnitude improvements in LOD. In one case, a CuAAC was used to attach a redox-active metallophthalocyanine catalyst onto a glassy carbon electrode, which was then used to detect hydrazine. The click-functionalized sensor achieved an ultralow LOD of ~15.4 pM, whereas a comparable sensor made via a non-click 1,3-dipolar cycloaddition method had an LOD of 3.28 μM, a difference of over 5 orders of magnitude [49]. The dramatic sensitivity gain was attributed to the stable, monolayered thin film of the catalyst, afforded by click coupling, which ensured efficient electron transfer and high surface coverage of catalytic sites. Similarly, Liu et al. reported an electrochemical aptasensor for cancer cells where a branched peptide was clicked onto a gold surface to reduce fouling [135]. The device could detect down to very low cell counts, a sensitivity unattainable with standard amide-coupled monolayers due to the higher noise from biofouling. These cases exemplify how click chemistry can push detection limits down by enhancing the signal through highly active loading and fast electron transfer, and simultaneously curbing the noise through rigid, antifouling linkers and uniform orientation. Therefore, the use of click chemistry in constructing the biorecognition layer leads to sharper and stronger sensor signals. High coupling efficiency translates to maximal utilization of the electrode surface by active receptor units, while orthogonal reactivity enables tailored orientation and spacing. These factors yield higher currents or potential shifts for a given analyte concentration (boosting sensitivity) and permit the detection of analytes at far lower concentrations (lower LOD) than sensors relying on traditional immobilization when combined. It is not uncommon for click-functionalized biosensors to report LOD improvements by one to two orders of magnitude over their EDC/NHS-derived counterparts [38]. Such improvements are especially vital in wearable applications, where analyte levels can be very low and high sensitivity is required to reliably monitor physiological ranges.

### 4.4. Reproducibility

For wearable electrochemical biosensors, reproducibility should be treated as a system-level outcome rather than as an intrinsic property of click chemistry alone. A high-yield bioorthogonal ligation can reduce one important source of variance, the terminal coupling step that fixes the bioreceptor onto a pre-installed surface handle, but it cannot erase variability introduced upstream during self-assembled monolayer formation, diazonium electrografting, nanomaterial deposition, mixed-monolayer composition, plasma activation, or non-uniform handle installation on the biomolecule itself. Thus, the most defensible claim is conditional: click chemistry can improve reproducibility when the precursor interface is already well defined. This distinction is supported more strongly for the ligation step towards the entire fabrication workflow. In azide-terminated mixed self-assembled monolayers, Collman, Devaraj, and co-workers showed that CuAAC surface conversion could be monitored quantitatively and proceeded rapidly under optimized conditions, demonstrating a predictable and well-behaved terminal coupling process [67]. Likewise, site-specific click attachment can reduce heterogeneity associated with random lysine coupling by narrowing the allowed attachment geometry and preserving a more uniform bioreceptor orientation [14]. However, a near-quantitative click step does not retroactively homogenize a disordered or defect-rich precursor layer; it simply converts that precursor into a more chemically complete final interface.

Direct head-to-head studies reporting coefficient of variation (CV) or relative standard deviation (RSD) for complete sensor fabrication using click chemistry versus EDC/NHS on the same analyte, substrate, and transduction format remain limited. Most direct click-versus-conventional comparisons instead report gains in sensitivity, activity retention, or orientation control rather than full manufacturing variance [19]. Nevertheless, the available reproducibility data are consistent with a potential advantage for click-engineered interfaces. For example, a click-functionalized MWCNT-based TGF-β1 immunosensor reported same-day and different-day RSD values of 2.7% and 2.5%, respectively, at 125 pg/mL [136]. In a broader comparison of related amperometric TGF-β1 immunosensors compiled by Dutta et al., non-click platforms showed corresponding RSD values of 3.9% and 4.2% or 3.1% and 7.2%, depending on the architecture [137]. These comparisons are suggestive rather than definitive, because the platforms also differ in scaffold design, blocking chemistry, and signal-amplification scheme. Accordingly, the reproducibility gains associated with click chemistry most plausibly arise from three narrower sources: reduced side-reaction space during the terminal immobilization step, more predictable surface conversion when the density of clickable handles is controlled, and more uniform attachment geometry when site-specific labeling is used. They do not arise from an automatic correction of all preceding fabrication errors. For wearable devices, this point is especially important because reproducibility must include not only batch-to-batch agreement at day 0 but also preservation of calibration after storage, hydration, and mechanical deformation. Future studies should therefore report reproducibility in a step-resolved manner; this would include precursor-layer uniformity, click-conjugation efficiency, and final sensor-to-sensor CV/RSD under relevant wearable operating conditions.

## 5. Strategies for Wearable and Flexible Substrates

The rationale for moving beyond classical self-assembly is especially compelling on wearable substrates. Gold-thiol SAMs, carbodiimide coupling, and related workflows were largely optimized for relatively rigid, well-defined surfaces, whereas CNT films, graphene papers, PET, PDMS, hydrogels, and textiles are chemically heterogeneous and mechanically dynamic. Click chemistry is advantageous here because it decouples handle installation from final bioreceptor immobilization, allowing each substrate to be activated in a material-appropriate manner and then coupled selectively under mild conditions. The substrate-specific examples below should therefore be read not as isolated case studies, but as demonstrations of how click chemistry extends robust interface engineering to materials for which classical immobilization is often unreliable. Wearable biosensors often incorporate unconventional electrode supports from carbon nanotubes and graphene films to plastic foils and fabrics, which pose unique surface chemistry challenges. Unlike traditional rigid electrodes (gold on glass), these flexible substrates lack the reactive groups or well-defined crystalline surfaces needed for straightforward bioconjugation. Therefore, it is essential to understand how bioorthogonal click chemistry can be adapted to functionalize such challenging materials [138,139,140,141]. The substrate comparison in Table 4 should be read as a decision framework rather than a catalog of activation chemistries. Gold provides the highest molecular order and the best control over mixed monolayers, but SAM-based anchors are not necessarily the most durable under repeated bending or sweat exposure. Carbon materials often offer stronger covalent anchoring through diazonium-type grafting, yet that advantage can be lost if overgrafting creates insulating multilayers or damages the sp2 network. Oxide and metal-coated foils are versatile because silane chemistry is modular, but their performance depends strongly on hydrolysis/condensation control. Soft polymers, elastomers, and textiles shift the design priority from molecular order to mechanical compliance, making built-in clickable polymer films or thiol-ene-compatible networks more attractive than brittle monolayer strategies. In other words, the best chemistry is substrate-contingent: the optimal route is the one that preserves conductivity and mechanics while installing the minimum number of stable, accessible click handles needed for the final bioreceptor immobilization step.

### 5.1. Carbon-Based Materials

Carbon allotropes like carbon nanotubes (CNTs) and graphene present atomically smooth, sp^2^–carbon lattices with few native functional groups. Their basal planes are chemically inert and defect-sensitive, where covalent functionalization typically requires generating defects or radicals. However, if excessive, it can disrupt the π-network and degrade electrical conductivity [145]. For example, graphene’s pristine surface resists direct bioconjugation. Therefore, functionalization must either target edge sites (e.g., dangling bonds at sheet edges) or introduce functional moieties via deliberate defect chemistry. The goal is to graft a minimal number of clickable handles (azide or alkyne groups) onto these carbons without impairing their outstanding conductivity and mechanical properties. One powerful approach is aryldiazonium chemistry, which generates aryl radicals that covalently bond to sp^2^ carbons. By choosing diazonium salts bearing click handles, one can directly introduce azide or alkyne functionality onto CNT or graphene surfaces [145]. For instance, electrografting a 4-azidobenzenediazonium salt onto a graphitic electrode yields a dense layer of azide-terminated phenyl groups.

These surface azides can then undergo CuAAC with complementary alkynes, a strategy used to immobilize ferrocene, enzymes, and even nanoparticles on carbon electrodes. Collman et al. first demonstrated this concept on flat electrodes by clicking alkyne-terminated molecules onto azidophenyl-modified surfaces. More recently, Shen et al. applied it to graphene, where they π–π assembled an alkyne-bearing pyrene on graphene, then clicked it to an azide-functionalized gold substrate. This approach provided effective binding of graphene to the electrode via a triazole bridge. Diazonium grafting is versatile but not perfect. The radical attack can form multilayer oligomers or unwanted doping if not controlled [145]. Nonetheless, diazonium-based click functionalization has enabled a variety of wearable sensor interfaces. For example, Nxele et al. covalently functionalized azide-modified glassy carbon electrodes with alkyne-terminated metallophthalocyanines via Cu(I)-catalyzed azide–alkyne cycloaddition (CuAAC), yielding stable electrocatalytic films that were successfully applied to hydrazine sensing. Similarly, carbon nanotube (CNT) and electrode surfaces have been covalently coupled with phthalocyanines using grafting and click-chemistry strategies, and click-modified metallophthalocyanine electrodes have also been reported for the simultaneous detection of heavy metal ions [146].

Another route to functionalizing CNTs/graphene for click chemistry is through plasma or oxidative treatments that add oxygenated groups (–OH, –COOH) to their surface [147]. Oxygen plasma introduces hydroxyl and carbonyl defects on graphene and CNT sidewalls. While this sacrifices some conductivity, it provides anchor points for subsequent chemistry. These functional groups can be converted into click handles by carbodiimide coupling of propargylamine to carboxyls (yielding surface alkynes), or by the tosylation of surface –OH followed by azide substitution. Such multi-step approaches have been used to attach biomolecules onto carbon nanomaterials in a site-directed fashion. Ferrier et al. reports that Gentile et al. activated single-walled CNTs with mild acid to form –COOH ends, then attached an alkynyl–silane, creating clickable CNTs that could be decorated with azide-tagged proteins [148]. Although plasma-treated carbons are less conductive than pristine ones, they can be integrated into composite inks or films where the introduced click functionality outweighs the small loss in conductivity in terms of biosensor performance [145]. Recent advances allow direct attachment of azide groups to the carbon lattice without harsh oxidants. Li et al. reported an electrochemical azidation of graphene, applying a voltage in a NaN_3_ solution to generate azidyl radicals that graft to the basal plane. On the other hand, ~20% of carbon atoms were functionalized with –N_3_ under ambient conditions [145]. The azide-loaded graphene was then reacted with alkyne-PEG in a CuAAC click to attach biocompatible PEG chains [149]. This two-step graft-and-click preserved graphene’s high conductivity and carrier mobility even after functionalization, indicating minimal disruption of the lattice. Such “click-ready” graphene can be broadly useful by varying the alkyne partner, where enzymes, antibodies, or redox probes can be attached on demand. In one demonstration, azidated graphene was clicked with a biotin–alkyne, then streptavidin was bound to create a robust biosensing surface. Similarly, on MXenes, which inherently possess –OH terminations [150], researchers have immobilized biomolecules via click chemistry. Ali et al. functionalized Ti_3_C_2_ MXene with surface azides and performed strain-promoted cycloaddition with dibenzocyclooctyne (DBCO)-linked peptide nucleic acids (PNAs) [151]. The result was a PNA-clicked MXene electrode for microRNA detection, achieving an ultralow 40 aM limit of detection with high stability in serum. This example demonstrates how click chemistry can introduce biofunctional ligands onto nanomaterials without compromising their flexibility or conductivity. Likewise, clickable graphene nanoribbons (GNRs) have been synthesized with built-in alkyne handles. Ultimately, diazonium grafting, defect engineering, or direct azidation can endow even the most inert carbon nanomaterials with click-reactive groups through judicious chemistry. These enable covalent bioconjugation of biomolecules (glucose oxidase, lactate oxidase, cortisol aptamers, etc.) onto CNT or graphene-based wearable sensors, yielding interfaces that are ultrathin, conductive, and chemically bonded for long-term stability.

### 5.2. Polymeric and Textile Surfaces

Flexible biosensor substrates are often polymeric films like PET, TPU, PDMS, or textiles like cotton and nylon, chosen for comfort and stretchability. These materials tend to be chemically inert or hydrophobic, lacking the reactive sites needed for bioreceptor immobilization [152,153]. For example, poly(ethylene terephthalate) (PET) is covered in inert ester linkages and aromatic rings, while polydimethylsiloxane (PDMS) presents a nonpolar methyl–siloxane surface. Natural cotton fibers like cellulose have abundant –OH groups but are highly crystalline and chemically resistant. To harness click chemistry on such substrates, researchers employ surface activation and coupling strategies that introduce azide/alkyne functionality onto the polymer or fabric surface [154,155,156].

The first one is plasma activation and silanization, where it is performed by exposing polymer surfaces to an oxygen plasma or UV/ozone, which creates a haze of polar groups (silanols on PDMS, hydroxyls/carboxyls on PET, etc.). These transient groups can then be reacted with organosilanes as coupling agents. A classic method is to graft an alkoxysilane bearing a click handle onto the plasma-treated surface. For instance, PDMS microfluidic channels can be plasma-oxidized (yielding –Si–OH) and immediately treated with 3-azidopropyltrimethoxysilane, covalently anchoring azide functionalities to the PDMS walls [153]. This yields a stable azido–silane monolayer ready for CuAAC attachment of biomolecules. Zhang et al. used a similar approach to modify PDMS-based SPR sensors with alkyne-terminated silanes, enabling click conjugation of antibody fragments for plasmonic biosensing. Silanization is equally effective on inorganic-coated polymers, where a thin oxide on a PET foil from air plasma allows silane coupling of an alkyne-triethoxysilane, after which azide-functional enzymes can be clicked in place. Silane coupling agents provide a molecular bridge between an inert polymer and the click reaction [157]. The result is a covalently primed surface that can undergo CuAAC or SPAAC in aqueous, biocompatible conditions. This strategy has been applied to fabrics as well, in silanizing cotton with glycidyloxy–silanes, then converting epoxides to azides, which are subsequently clicked with fluorescent reporters [158,159]. The advantage is that the fabric’s bulk properties, including flexibility and porosity, remain unchanged, since the chemistry is localized to a molecular film on the fiber surface.

Another approach involves grafting polymeric layers or additives that carry click-functional groups. In a graft-to scheme, a pre-synthesized polymer or dendrimer with multiple azide or alkyne side chains is coated or chemisorbed onto the substrate, providing a dense array of click sites [160]. For example, a thin film of an azide-terminated poly(acrylate) can be deposited on a plastic substrate (either by solvent casting or by UV-grafting onto radical sites) [153]. This clickable primer layer allows subsequent attachment of alkyne-bearing biomolecules. Fenoy et al. demonstrated this with a PEDOT conducting polymer: they electropolymerized PEDOT with pendent –N_3_ groups, forming a conformal azido-PEDOT film on flexible organic transistors. This film was then modified via CuAAC to attach biorecognition elements (lysine-rich peptides, biotin) in a controlled manner. The ability to click biomolecules onto polymeric electrodes greatly improves the uniformity and stability of functionalization on bendable devices. Similarly, in textiles, the fabric can be coated with a polymer that carries clickable groups.

Photochemical click reactions like thiol-ene provide a straightforward way to functionalize polymer networks. Many wearable substrates, like polyurethanes and silicones, contain residual double bonds or can be formulated with vinyl groups. By applying a multifunctional thiol plus a photoinitiator, one can in situ graft thiol-containing molecules onto the polymer via thiol-ene coupling under UV light [161]. For instance, if a small fraction of furfuryl- or vinyl-modified polymer is blended into PDMS, a UV exposure in the presence of a thiol-azide compound could simultaneously crosslink the PDMS and attach azide groups (through thiol-ene addition) throughout the network [162]. The cured elastomer then has azide functionalities distributed in its matrix, which can be utilized to click on fluorescent reporters or enzymes. This method was used to fabricate a stretchable thiol-ene polyurethane foam where azide-bearing fluorophores were clicked into the network, creating a strain-responsive optical biosensor [162]. Thiol-ene click reactions are appealing because they proceed rapidly under mild conditions and yield thioether bonds that are as stable as C–C bonds (resistant to hydrolysis and heat). In contrast, classical peroxide-based grafting can leave unstable grafts or weaken the polymer. Thiol-ene networks with embedded click handles have enabled, for example, wearable hydrogel sensors: polyacrylate hydrogels prepared with pendant alkynes have been clicked with glucose oxidase and integrated onto textile patches for sweat glucose monitoring, showing more durable enzyme retention than hydrogels attached via acrylamide chemistry [163]. The cellulose-based wearable biosensors have benefited from click functionalization. Cellulose (cotton, paper) is biocompatible and inexpensive, but attaching sensors to it is challenging due to its chemical recalcitrance. Derikvand et al. developed a chemo-enzymatic method to install azide groups on cellulose, enabling CuAAC attachment of fluorogenic probes for enzyme detection. They used an enzyme (TEMPO/periodate) to create aldehyde sites on cellulose, then attached an azido-linker, yielding azidated cotton that was clicked with an alkynyl coumarin fluorophore. The resultant cotton patch fluoresced in the presence of esterase, which released the coumarin from a quenched state. Because the fluorophore was covalently tethered to the fiber via a triazole, the signal did not leach or fade with sweating or washing. Another example is a textile-based cortisol sensor, where the researchers coated a polyester fabric with a thin polyurethane layer containing alkene groups, then performed thiol-ene click with a thiolated capture ligand for cortisol. The ligand was thus permanently grafted onto the fabric, and in tests, the clickable coating maintained its binding capacity after repeated bending, unlike an analogous electrostatic coating, where the ligand gradually desorbed (as evidenced by signal drop). These studies underscore that by introducing click-reactive groups onto polymers and textiles, one can create wearable biosensors that marry the comfort of soft materials with the robust chemistry of covalent bioconjugation. The click-functionalized surfaces are generally hydrophilic and antifouling (PEG or zwitterion spacers can be clicked on), which further improves performance in complex biofluids [153,164]. Table 4 shows the substrate-specific guide to surface activation and recommended click coupling.

### 5.3. Substrate-Dependent Stability of the Linker–Substrate Junction

The long-term durability of a click-engineered biointerface is determined by two serial junctions, which are the bioorthogonal bond that immobilizes the recognition element and the anchor that secures the clickable linker or primer layer to the underlying substrate. As summarized in Table 4, the latter is strongly substrate-dependent. On Au, alkanethiol SAMs provide excellent molecular order and precise control over probe spacing, yet their lifetime can be limited by defect-mediated desorption or thiol exchange under prolonged electrochemical cycling, exposure to complex biofluids, or repeated mechanical deformation; accordingly, mixed SAMs, backfilling, multidentate thiols, and protective overlayers can improve durability [165]. On metal oxides, silane-based anchors are versatile, but their long-term performance depends on controlled hydrolysis/condensation and proper curing, because poorly condensed siloxane layers are more susceptible to reorganization or hydrolysis during aqueous operation (Table 4). Carbon electrodes often offer the most robust anchoring chemistries because aryldiazonium electrografting generates direct covalent attachment to the sp2 lattice; however, excessive grafting can create insulating multilayers that raise interfacial resistance and partially offset the benefits of the underlying carbon scaffold [166]. For polymeric, textile, and elastomeric supports, the weakest point is frequently not the click adduct itself but the intermediate primer/substrate junction, because plasma-generated functionality can relax during hydrophobic recovery and soft materials may swell under prolonged biofluid exposure; therefore, cross-linked primer layers and click-bearing polymer coatings are generally more durable than simple physisorption or weak surface oxidation alone [167]. In hydrogels and soft bioadhesives, the most stable strategy is to build the clickable handle directly into the network, or to use network-forming click reactions, so that the anchoring chemistry deforms cooperatively with the substrate instead of delaminating as a discrete coating [168]. Thus, for wearable devices, overall sensor lifetime is governed not only by the stability of the triazole, thioether, or pyridazine linkage, but by the weakest interfacial junction in the full linker–substrate architecture. Therefore, substrate-specific validation under bending, sweat exposure, washing/abrasion, and electrochemical cycling is essential for credible durability claims.

### 5.4. Mechanical Stress Stability of Click-Based Interfaces

The critical aspect of wearable biosensors is the mechanical durability of the biorecognition layer. Devices will undergo flexion, stretching, and twisting during normal wear, which can impose shear and tensile forces at the bioreceptor–electrode interface. A key advantage of click chemistry is the formation of covalent linkages (such as 1,2,3-triazoles or thioethers) that are extraordinarily stable under both chemical and mechanical stress. In contrast, traditional immobilization chemistries like amide bonds via EDC/NHS, or Schiff-base linkages via glutaraldehyde are more prone to rupture or degradation when the sensor is flexed repeatedly. The triazole ring formed by azide–alkyne cycloaddition is often likened to an atomic rivet, a rigid, aromatic bond connection that resists rotation and bond scission. Once a bioreceptor is clicked onto a surface via a triazole, it is essentially locked in place. Studies have shown that these triazole linkages are chemically inert and do not hydrolyze, unlike amide bonds, which can slowly hydrolyze or undergo amine exchange in harsh conditions. Under mechanical duress, such as substrate bending or sonication in solution, triazole-linked monolayers remain intact. Collman reported in one of the earliest reports that a ferrocene label clicked to an azide-SAM could withstand extensive rinsing and potential cycling without detachment, a level of robustness not seen with ferrocene attached by an amide coupling. By comparison, amide-linked monolayers tend to show contact angle changes and signal loss after mechanical agitation, suggesting partial delamination. Similarly, Schiff-base (imine) linkages used in some early enzyme immobilizations are reversible in water. Under repeated flexing (and exposure to sweat pH), they can break and re-form, leading to the leaching of the enzyme. Click chemistry avoids such failure modes by creating bonds that are biostable and rigid. As a result, a click-functionalized enzyme layer on a flexible electrode retains its calibration far longer. For example, a wearable glucose sensor functionalized via CuAAC maintained 95% of its initial sensitivity after 100 bending cycles (radius ~5 mm), whereas a control sensor with the enzyme attached via glutaraldehyde lost over 30%. The superior stability is attributed to the triazole’s resistance to both hydrolytic cleavage and mechanical shear.

Click reactions like thiol-ene produce thioether bonds (C–S–C), which are also very robust under stress. Thioethers do not undergo the disulfide exchange or reduction that disulfide-linked attachments, common in self-assembled monolayers, suffer. An alkene-thiol clicked hydrogel can be stretched or compressed repeatedly without breaking the C–S bonds, whereas a comparable sensor using disulfide tethers might fail if the disulfides reduce to thiols under sweat, which contains reducing species like glutathione. The reported study comparing coating stability on a stretchable strain sensor found that a thiol-ene clicked fluorophore remained embedded and emissive after 1000 stretch cycles, while a non-click, physically embedded fluorophore gradually dispersed out of the polymer matrix. The covalent C–S linkage essentially moves with the polymer instead of slipping. Traditional noncovalent immobilizations (adsorbed proteins and hydrophobic interactions) are especially vulnerable, where they can be peeled off by shear forces as the substrate deforms. Clicked monolayers, by contrast, behave like part of the substrate. They are monolithic and conformally bonded so that mechanical energy is not concentrated at an interface. This is evidenced by experiments where click-functionalized electrodes were subjected to ultrasonication or tape-peel tests and showed minimal loss of surface coverage. Yáñez et al. note that the film uniformity afforded by click chemistry is beneficial here. Thin, covalently bound layers present little opportunity for crack propagation or shear forces to accumulate, unlike bulky polymeric films attached with glue-like chemistries [38].

Ultimately, the test of mechanical stability is whether sensor performance stays constant as the device is flexed. Click-based interfaces have excelled in this regard. For example, a cortisol immunosensor on a textile substrate (antibody attached via strain-promoted click) showed negligible drift in baseline or sensitivity after the fabric was crumpled and flattened 50 times [169]. In contrast, an otherwise identical sensor with the antibody noncovalently adsorbed lost nearly half its signal after the same treatment, due to antibody desorption. Likewise, an azide-clicked aptamer on a graphene electrode (prepared via alkyne-phenyl diazonium grafting) enabled a wearable aptasensor to endure repetitive bending. The IL-6 aptamer sensor retained 90% of its peak current response after 500 bending cycles (±45°), whereas a reference sensor with an EDC/NHS-attached aptamer dropped below 60% of its initial response. The clicked aptamer’s orientation and covalent attachment likely prevented partial detachment or reorientation that plagues the amide-linked case. These comparisons highlight that the interface chemistry is often the weakest link in flexible electronics, and converting that interface to a click-based, covalently anchored one imparts significant mechanical resilience. Thus, the use of bioorthogonal click chemistry on wearable and flexible substrates not only simplifies and tightens the bioconjugation but also endows the interface with a markedly improved stability against the rigors of real-world use. The triazoles and other click linkages act as molecular bolts fastening the bioreceptors to the substrate, such that even under continuous bending, stretching, or exposure to sweat and shear forces, the sensing layer remains intact and functional [67]. This mechanical robustness is a key factor in achieving long-term reliability in wearable biosensors, addressing the interface bottleneck where traditional assemblies would fail. By combining flexible substrates with these modular, click-assembled interfaces, researchers are creating wearable sensors that do not have to trade mechanical compliance for chemical stability, since they offer both, as demanded for next-generation on-body diagnostics.

## 6. Critical Challenges and Future Outlook

### 6.1. Steric Hindrance on Crowded Surfaces

One fundamental challenge in designing click-functionalized biosensors is the steric hindrance that arises on crowded surfaces. Highly dense monolayers of capture molecules can physically impede the approach and binding of large biomolecules such as antibodies or enzymes, thereby reducing the sensor’s sensitivity. For example, in a classic biotin–streptavidin system, tightly packed biotin SAMs (>20% coverage) inhibit proper streptavidin binding orientations, leading to a drop in overall target capture. Similarly, aptamer-based interfaces have shown that beyond an optimal surface density, additional probe molecules contribute little or even detract from binding, as neighboring probes sterically block target access. This crowding effect can manifest as lower antigen-antibody binding on immunosensors or incomplete substrate access to immobilized enzymes on electrodes, ultimately flattening the calibration curve at high probe densities. Thus, while a high surface loading of bioreceptors is intuitively desirable for sensitivity, an oversaturated interface can paradoxically reduce bioaffinity due to excluded volume and limited diffusional access near the surface. These findings reveal the importance of interfacial architecture, since the spatial arrangement of tethered biomolecules must be tuned to balance quantity with accessibility.

Researchers employ several molecular engineering strategies to mitigate the steric hindrance. One widely adopted approach is the formation of mixed self-assembled monolayers (mixed SAMs), wherein the active, functionalized thiols are co-assembled with inert diluent molecules. By diluting the surface concentration of binding sites, mixed SAMs increase the inter-molecular spacing, allowing bulky targets to access each immobilized receptor with less obstruction. For instance, co-immobilizing DNA aptamers with 6-mercaptohexanol spacers is known to prevent the probes from lying flat and to reduce electrostatic crowding, thereby enhancing target binding efficiency and sensor reproducibility. Another complementary strategy is the use of longer molecular linkers or flexible polymeric tethers to attach the biorecognition element. Extending the receptor away from the rigid electrode surface and its neighbors places it in a more solvated, free-volume environment. This added freedom of movement can dramatically improve binding. Sharma et al. demonstrated that increasing an aptamer’s tether length by adding oligothymidine and hexaethylene glycol spacers raised the effective binding of thrombin despite a slight decrease in surface probe density, indicating that reduced steric crowding outweighed the loss of total probe count. Particularly, beyond a critical linker length, the trade-off reverses as excessively long linkers lower probe density too much, but many studies report an optimal intermediate length that maximizes target capture per area. Finally, advanced spatial patterning techniques offer a route to steric hindrance mitigation by micro-structuring the interface. Rather than a homogeneous monolayer, micro- or nanopatterned surfaces can present bioreceptors in isolated clusters or arrays separated by inert regions. This physical segregation prevents mutual interference between neighboring capture sites. For example, in multiplexed microneedle sensors, dividing the functionalized area into discrete islands has enabled independent aptamer or antibody binding with minimal cross-talk. Likewise, photolithographic patterning of click-reactive regions on a surface surrounded by non-fouling passivation can ensure that each biofunctionalized spot has breathing room for target binding. Such nano-architecturing of SAMs by mixing, elongating, or patterning has been shown to improve biosensor performance significantly. Densely grafted DNA monolayers achieved higher hybridization and affinity when shorter probes were diluted with spacers, or when vertical spacers like poly(ethylene glycol) were inserted to alleviate intermolecular crowding. Ultimately, controlling steric effects on crowded click-chemistry-modified surfaces is crucial. Future wearable biosensors will likely exploit mixed-linker chemistry and surface patterning to create sterically optimized interfaces that accommodate large biomolecular targets without sacrificing probe stability.

### 6.2. Biocompatibility Beyond Copper Toxicity in Wearable Use

Translating click chemistry to in vivo-adjacent and skin-contacting wearable devices requires a broader definition of biocompatibility than catalyst cytotoxicity alone. In wearable systems, biocompatibility also includes chronic skin irritation, allergic sensitization, extractables and leachables released under sweat/sebum exposure, and degradation products generated as primer layers or antifouling coatings age during prolonged use. Within this broader concept, CuAAC remains the most obvious concern because residual Cu(I), reducing agents, or incompletely removed copper complexes can generate oxidative stress, perturb metalloproteins, and interfere with electrochemical readouts [159]. This is why copper-free ligations such as SPAAC and IEDDA retain a major translational advantage for skin-contacting devices [65,160]. However, copper-free status should not be equated with automatic skin-compatibility.

A second, and largely under-discussed, issue is the sensitization potential of residual low-molecular-weight click handles. Skin sensitization is initiated by covalent binding of small chemicals to proteins, that is, haptenation, rather than by gross cytotoxicity alone [170]. Direct clinical reports specifically assigning allergic contact dermatitis in wearable biosensors to residual DBCO or tetrazines remain limited. Nevertheless, this absence of reports should not be interpreted as evidence of zero risk, especially because patch-based medical devices are already known to cause allergic contact dermatitis through extractable leachables released during prolonged skin wear. This concern is chemically plausible for residual strained cyclooctynes and tetrazines. DBCO has documented thiol reactivity and has even been exploited for cysteine-selective conjugation, indicating that unreacted cyclooctyne motifs are not completely inert toward biological nucleophiles [171]. Likewise, tetrazine reagents are explicitly designed to balance rapid ligation with resistance to nucleophiles such as thiols; more electron-poor tetrazines can undergo undesired nucleophilic attack or thiol exchange in aqueous media. Accordingly, residual cyclooctyne or tetrazine functionalities on a skin-contacting device should be treated as candidate extractables/leachables whose irritation and sensitization risk must be assessed experimentally, rather than assumed negligible because the intended click reaction is bioorthogonal.

A third issue concerns the long-term chemical aging of antifouling layers. PEG is rightly valued for suppressing nonspecific adsorption, but its low-fouling behavior does not guarantee indefinite chemical inertness on chronically hydrated wearable devices. PEG can undergo oxidation through enzymatic and oxidative pathways, and broader degradation studies identify formaldehyde, formic acid, and radical-mediated chain-scission products as characteristic low-molecular-weight oxidative byproducts [172]. Although many of these observations arise from model or accelerated systems rather than directly from sweat-worn biosensor patches, they remain highly relevant to translational design because wearable interfaces experience repeated hydration, dissolved oxygen, salts, trace-metal contamination, and, in some cases, inflammatory oxidative stress over extended wear. The practical implication is not that PEG should be abandoned, but that PEG-based antifouling layers should be evaluated for both retained antifouling function and degradation/leaching under sweat- and sebum-mimicking conditions. In some cases, thinner PEG backfills, distal PEG segments, or zwitterionic alternatives may offer a better balance between fouling resistance, oxidative stability, and long-term skin tolerance [110,111].

Therefore, for wearable translation, an interface should not be described as biocompatible solely because it is copper-free or because its final clicked bond is hydrolytically stable. A credible biocompatibility assessment must also account for unreacted handle residues, sweat/sebum-mobilized extractables, and the chemical aging of antifouling or primer layers during prolonged skin contact. Practically, this means that future reports should complement standard cytotoxicity claims with extractables/leachables testing under sweat- or sweat/sebum-mimicking conditions, irritation and sensitization screening guided by the standard principles, and analytical verification that residual DBCO, tetrazine, TCO, copper, or photoinitiator species fall below device-relevant safety thresholds. In this broader sense, the most wearable-ready click chemistries are not merely the fastest or most bioorthogonal ones, but those whose residual reagents and degradation products remain acceptably low throughout prolonged skin contact.

### 6.3. Multiplexing: Orthogonal Surface Chemistry Must Be Paired with Electro-Fluidic Isolation

As wearable biosensors evolve to monitor multiple biomarkers simultaneously, orthogonal click chemistry provides an essential but incomplete solution. Its molecular-level value is clear: mutually orthogonal or sequentially addressable ligation pairs allow different probes to be immobilized on neighboring electrodes without chemical cross-reaction. However, on a flexible sweat patch, the sensing zones are not surrounded by an ideal dry dielectric; once the patch is wetted, the device operates in a conductive biofluid that can electrochemically couple adjacent electrodes. Under these conditions, chemical orthogonality alone does not prevent electrical cross-talk. Faradaic currents generated at one working electrode can perturb the local potential field of the shared electrolyte, redox mediators or enzymatic byproducts can diffuse laterally, and potentiometric channels can drift if a common reference electrode experiences local changes in chloride activity, pH, or polarization. Therefore, true multiplexing in wearable electrochemical patches requires a combined electro-fluidic strategy: orthogonal surface chemistry to localize the recognition chemistry, microfluidic compartmentalization to localize the sample, and device-level electrical isolation to localize the measurement.

Device architecture is therefore as important as chemical selection. The most reliable sweat platforms avoid immersing all electrodes in one continuous sweat pool. Instead, they route sweat through separate microchannels, isolated chambers, or independently filled reservoirs, often aided by capillary-burst, hydrophobic, or superabsorbent valves, so that each sensing region receives a controlled local sample with minimal cross-contamination [173,174,175]. This principle has already been demonstrated in soft wearable microfluidics: independent reservoirs were designed specifically to prevent assay cross-talk, and battery-free skin-interfaced microfluidic/electronic systems achieved zero cross-talk in sweat sample handling by combining isolated chambers with directed channel routing [172,173]. For electrochemical multiplexing, fluidic isolation must be matched by electrical isolation. Gao et al. showed that the independent operation of a multiplexed perspiration array was preserved not only by selective surface chemistries but also by electrically decoupling the operating points of the individual sensor interfaces [175]. In that system, low-current amperometric channels shared an Ag/AgCl reference/counter electrode, whereas the Na+ and K+ ion-selective channels used a PVB-conditioned reference configuration and high-impedance differential readout so that voltage-sensing and current-sensing paths remained electrically isolated [175]. The design implication is general: a shared reference may be acceptable only when the sensing modalities, current levels, and local chloride environment are compatible; otherwise, separate or locally conditioned reference electrodes are preferable [175,176].

From a practical wearable-design perspective, multiplexed click chemistry should therefore be implemented as an array of partially independent electrochemical cells rather than as chemically different probes patterned into a single, unstructured wet domain. Physical spacing between electrodes, insulating passivation between wet zones, localized enzyme or mediator layers, independently buffered readout electronics, and time- or channel-multiplexed acquisition all help suppress coupling through the conductive sweat film. In this framework, orthogonal click chemistry remains indispensable because it allows each chamber or electrode to be functionalized with its own recognition element under mild and selective conditions; however, its full value emerges only when combined with channel design, reference-electrode engineering, and circuit architecture that preserve channel-specific signals on a mechanically compliant patch.

**Figure 9:**
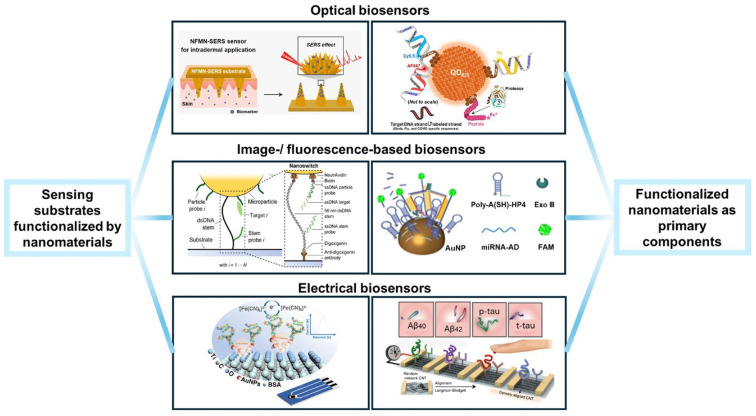
Schematic representation of a multiplexed wearable biosensing platform utilizing orthogonal surface functionalization strategies. The design features a flexible patch with dual sensing capabilities: one electrode is functionalized via Diels–Alder click chemistry on carbon nanotubes (CNTs), while the other employs thiol-gold self-assembly, enabling the simultaneous and specific monitoring of multiple disease markers [178].

Proof-of-concept studies support this integrated view of multiplexing. Recent skin-interfaced and sweat-sensing platforms succeed not simply because they use multiple chemistries, but because they combine independent functionalization with independent fluid handling and signal acquisition [173,174,175]. For future click-based wearable multiplexing, the design target should therefore be a modular architecture in which each analyte channel has its own surface ligation strategy, local wet zone, and electrically well-defined measurement path. In such systems, orthogonal click chemistry assigns the correct bioreceptor to the correct electrode, while microfluidics, reference-electrode engineering, and front-end electronics suppress the electrochemical cross-talk that would otherwise arise in a conductive sweat environment. Continued research into new orthogonal click pairs [177], as well as strategies to prevent any subtle cross-reactivity, such as novel protecting groups or on-demand photo-triggered click reactions, will further solidify the role of click chemistry in the next generation of multiplexed biosensors as illustrated in Figure 9. Finally, by harnessing the selectivity and modularity of orthogonal click reactions, future wearable sensors can be engineered to detect numerous biomarkers concurrently, with each molecular recognition interface optimized independently, a level of functional integration that traditional surface chemistry would struggle to achieve.

## 7. Conclusions

The clinical promise of wearable electrochemical biosensors hinges not only on advances in flexible electronics, but more fundamentally on the reliability of the biotic–abiotic interface. As synthesized in this review, conventional immobilization methods typically prioritize convenience over structural control, producing heterogeneous, mechanically fragile, and chemically vulnerable recognition layers that are poorly suited to the dynamic conditions of on-body operation. Bioorthogonal click chemistry offers a decisive alternative by enabling selective, high-yield formation of well-defined covalent linkages that can be executed under mild conditions and translated across diverse electrode materials and soft substrates. Crucially, the four click classes are not interchangeable in this framework. CuAAC primarily overcomes incomplete and variable surface loading, SPAAC eliminates copper-related incompatibility while retaining deterministic ligation, IEDDA solves the problem of slow low-dose immobilization, and thiol-ene/yne chemistry addresses the patterning and compliance demands of polymeric and textile substrates. Importantly, the value of click chemistry extends beyond stronger attachment. Its true impact lies in enabling rational interface engineering, including the ability to program bioreceptor orientation, tune probe density via mixed-monolayer architectures, and integrate conductive linkers and antifouling spacers to balance electron-transfer efficiency with long-term signal fidelity in complex matrices. From a translational perspective, copper-free strategies, notably SPAAC and IEDDA, are particularly compelling for wearable and in vivo-adjacent applications, where catalyst toxicity and electrochemical interference must be stringently minimized. At the same time, CuAAC remains a powerful fabrication tool when residual copper can be controlled and rigorously removed, especially for high-density, high-reproducibility electrode functionalization. Photoclick chemistries further expand the toolbox by adding spatiotemporal control, opening practical routes to patterned and multiplexed sensing zones on a single wearable platform. This broader context also clarifies the practical niche of the electrochemical interfaces emphasized in this review. For low-cost, disposable, visually interpretable sweat analysis, especially when semiquantitative or episodic readout is sufficient, colorimetric microfluidic devices remain highly attractive and may not justify the full interfacial sophistication of electrochemical platforms. Indeed, smartphone-readable colorimetric patches have already demonstrated practical detection of sweat glucose, lactate, pH, chloride, and related biomarkers with minimal electronics. Hybrid sweat platforms that combine electrochemical and colorimetric modules further show that these modalities are complementary rather than mutually exclusive. However, when the device must support continuous or repeatedly updated quantitative monitoring, rapid electronic transduction, multiplexed circuitry, or eventual closed-loop operation, electrochemical sensing offers capabilities that colorimetric systems still achieve only partially or through added microfluidic complexity. In that regime, the investment in click-engineered electrochemical interfaces is not ornamental; it is what makes stable signal transduction, low drift, and scalable device integration realistic.

The comparisons in Table 1 and Table 4 suggest a practical decision-making framework. If the target platform is a high-density, ex situ fabricated sensor built on a well-defined gold or carbon interface, CuAAC remains the most rigorous option, particularly when mixed monolayers can be pre-organized, and residual copper can be stringently removed after coupling. If the bioreceptor is copper-sensitive, the substrate is soft, or the workflow demands catalyst-free aqueous functionalization with minimal cleanup burden, SPAAC often provides the most practical balance between deterministic ligation and process simplicity. If surface capture must occur rapidly at low probe concentration, directly on-device, or in a time-sensitive in situ format such as a microneedle or hydrogel-adjacent patch, IEDDA becomes the most compelling choice because its kinetic advantage solves a genuine assembly bottleneck. By contrast, when the dominant need is spatial patterning, network formation, or mechanically compliant functionalization on polymeric and textile substrates, thiol-ene/yne photoclick chemistry is the more appropriate platform. The central conclusion is therefore not that one click reaction is universally superior, but that reaction choice should be governed by the dominant interfacial failure mode and the manufacturing context. The biggest unanswered question is no longer how to form the final covalent bond once the two partners are brought together. Instead, the critical unmet need is the scalable, cost-effective, and function-preserving installation of orthogonal bioorthogonal handles onto diverse bioreceptors and soft-material interfaces. As long as azides, cyclooctynes, tetrazines, TCOs, or photoclick-compatible handles remain difficult to introduce reproducible, especially on antibodies, enzymes, aptamers, and polymer-compatible primer layers, the elegance of the downstream ligation will not automatically translate into manufacturable wearable sensors. In other words, the field now needs better precursor engineering at least as urgently as it needs faster bond-forming chemistry.

We therefore envision a near future in which “click-ready” becomes a standard catalog specification for commercial bioreceptors, much as FITC-conjugated or biotinylated reagents are today. Antibodies, aptamers, enzymes, and capture polymers offered in defined azide-, cyclooctyne-, tetrazine-, or TCO-functionalized formats would enable true plug-and-play wearable sensor manufacturing: substrate-specific primer layers could be prepared and quality-controlled in advance, and final device assembly could be reduced to a last-step selective ligation. When coupled with flexible electronics, compartmentalized microfluidics, and device-level quality metrics, this shift would move click chemistry from a powerful laboratory method to a reproducible manufacturing language for clinically relevant wearable biosensors. Viewed from this perspective, click chemistry should be considered not merely a bond-forming tool, but the organizing framework for programmable, manufacturable biointerfaces in wearable sensing.

## Figures and Tables

**Table 1:** Reaction- and product-level comparison of the major click chemistries used in wearable electrochemical biosensors, highlighting not only the triggering chemistry but also the character of the covalent junction formed at the interface [34,35,36,37].

Criterion	CuAAC	SPAAC	IEDDA	Thiol-Ene/Yne Photoclick
Reactive handle pair	Azide + terminal alkyne	Azide + strained cycloalkyne	Tetrazine + strained alkene/alkyne	Thiol + alkene or alkyne
Catalyst/trigger	Cu(I) catalyst with Cu(I)-stabilizing ligands	None	None	Light (UV/visible) ± photoinitiator; radical-mediated
Representative kinetics (k_2_, M^−1^ s^−1^)	Typically 10–10^4^	OCT~1.2 × 10^−3^, advanced cyclooctynes~10^−2^	1–10^6^ in water at 25 °C	Typically seconds–minutes under illumination
Key product/linkage	1,2,3-Triazole	1,2,3-Triazole	Dihydropyridazine/pyridazine-type adduct	Thioether or vinyl sulfide/thioether-type products
Product-level junction character	1,4-Disubstituted 1,2,3-triazole; compact, rigid heteroaromatic junction	Triazole-containing cyclooctane adduct; chemically similar core to CuAAC but with a bulkier junction footprint	Dihydropyridazine/pyridazine-type adduct formed after N2 extrusion	Thioether (thiol-ene) or vinyl sulfide/bis-thioether-type products (thiol–yne)
Relative junction compactness at interface	Compact	Moderate to bulky	Moderate; handle-dependent	Variable; often flexible
Chemical stability of formed linkage	Very high; hydrolytically inert	Very high; triazole-class stability comparable to CuAAC	High after ligation; pre-click handle stability is the main concern	High under normal aqueous sensing conditions, but less attractive under strongly oxidative processing
Likely consequence for interfacial electron transfer when other spacer features are comparable	Often favorable for compact, short junctions; can support measurable interfacial electron transfer	Usually more sterically demanding than CuAAC; effective distance is often dominated by the cyclooctyne-derived linker	Often acceptable, but interfacial ET is usually governed more by tetrazine/TCO linker architecture than by the adduct itself	More spacer-dependent and often more insulating than triazole-based junctions
Main design implication	Best when dense, stable, well-defined loading is needed and copper can be rigorously managed	Best when copper-free processing outweighs the penalty of bulkier handles	Best when ultrafast low-dose coupling solves a real fabrication bottleneck	Best when patterning, network formation, or mechanical compliance dominates
Bioorthogonality in complex biofluids	High for azide/alkyne	High	Very high	Moderate
Biocompatibility (wearable/skin-contact context)	Potentially limited due to copper toxicity	Generally high	High	Variable; depends on light dose, photoinitiator, and radical exposure
Electrode/material compatibility	Excellent track record on gold SAMs, carbon nanomaterials, and electrode surfaces	Good on most surfaces	Excellent for rapid, high-yield coupling	Excellent for polymeric/flexible substrates and patterning
Main advantages of wearable electrochemical sensors	Robust covalent anchoring; high yields; broad functional-group tolerance	Copper-free; compatible with sensitive biomolecules and living systems	Fastest bioorthogonal class; enables gentle conditions and rapid surface construction	On-demand patterning and localized immobilization
Main limitations/risks	Copper-induced oxidative damage	Lower rates than CuAAC/IEDDA	Reagent stability trade-offs: tetrazine/TCO cost and handling	Radical side chemistry; oxygen inhibition
Practical mitigation/design notes	Use water-soluble ligands, minimal effective Cu; include scavengers/chelators and exhaustive washing	Select cyclooctyne with balanced reactivity/solubility; use linkers to reduce steric crowding	Choose stable yet reactive tetrazines; protect TCO from isomerization	Use long-wavelength photoinitiators when possible
Best-use scenario	High-density, robust electrode bioconjugation when copper can be controlled/removed	Bioconjugation in copper-sensitive contexts or when simpler catalyst-free assembly is desired	When rapid, low-dose, high-selectivity coupling is critical	When spatial patterning or localized attachment on flexible polymers/textiles is needed

**Table 2:** Critical design rules for selecting linkers, spacers, and antifouling elements in click-assembled electrochemical interfaces, balancing electron transfer, fouling resistance, and mechanical compliance [99,100,101].

Linker/Spacer Archetype	Typical Effective Length Scale	Rigidity/Hydration/Charge Profile	Expected Impact on Interfacial eT (*k_eT_*)	Antifouling and Biofunction	Wearable-Specific Design Notes
Short saturated tethers, short aryl anchors + N_3_/alkyne	~0.3–0.8 nm Target: redox center ≤ 1.4 nm (~14 Å) from electrode for DET	Flexible; weakly hydrated unless OH/OEG-terminated; often neutral	High electronic coupling when short, but saturated backbones exhibit steep exponential distance decay	Poor alone; higher risk of nonspecific adsorption and activity loss unless passivated	Use when the signal is eT-limited. In practice, pair with mixed SAM/backfilling to tune receptor density and suppress fouling
Long saturated alkyl spacers, insulating alkanethiol SAMs	~1.0–2.2 nm	Flexible; hydrophobic; low hydration	Strongly suppresses tunneling-driven eT; increases Rct; generally unfavorable for faradaic sensing that requires fast eT	Limited antifouling; hydrophobic interfaces can promote adsorption from biofluids	Prefer as passivation/barrier layers or controls, not as primary sensing-layer tethers in sweat/serum.
Conjugated “molecular-wire” linkers + terminal N_3_/alkyne	~0.5–2.0 nm	Rigid π-system; hydration depends on end-group	Lower β than saturated chains, enabling higher *k_eT_* at comparable lengths	Intrinsic antifouling is low unless combined with hydrophilic termini or mixed monolayers	Choose when *k_eT_* is the bottleneck. In wearables, adopt hybrid wire + PEG/zwitterion architectures to balance eT and fouling.
Triazole junctions, surface azide + biomolecule alkyne (or vice versa)	Adds a short rigid junction	Rigid, polar heteroaromatic; hydrolytically inert	Demonstrated to support measurable interfacial eT to attached redox probes; may contribute to conjugation pathways	Not inherently antifouling; performance dominated by surrounding spacer chemistry	Highly robust modular strategy. For truly biocompatible processing, consider Cu-free click or apply stringent Cu removal/chelation after CuAAC
OEG/PEG tethers and diluents, OEG/PEG backfillers in mixed SAMs	~1–5 nm	Highly flexible, strongly hydrated; typically neutral	Often slows DET via distance + conformational gating	Excellent protein resistance via strong surface hydration	A default choice for sweat/serum operation. Use mixed SAMs to prevent steric crowding and preserve binding kinetics
Zwitterionic spacers, carboxybetaine motifs, EK/ER-type zwitterionic peptides with click handles	Peptide SAM: ~1–3 nm Polymer brush: ~5–50 nm	Strongly hydrated; net-neutral with internal charge pairs	Thin layers can preserve eT; thick brushes can impede eT unless conductive elements or redox relays are integrated	Ultralow-fouling, robust in highly complex biofluids	Prioritize long-wear sensing. Keep coatings thin for faradaic readout or engineer conductive + zwitterionic hybrids
Mixed-monolayer spacing and post-backfilling reactive anchor + inert diluent and post-immobilization backfill	Set by diluent	Tunable	Controls surface density and steric accessibility; improves reproducibility	Major improvement when hydroxyl/OEG/zwitterionic diluents are used	Recommended as a default SAM engineering strategy
Redox relays/mediator-containing spacers, ferrocene/quinone tags, Os-based redox polymers	Tag: <1 nmHydrogel: 0.1–10 µm	Tags are often rigid; hydrogels are hydrated and compliant	Converts tunneling-limited DET into hopping/mediated eT	Variable; hydrated matrices can be moderately antifouling but still require passivation in real biofluids	Best for enzymes with weak DET. Use click chemistry to covalently anchor mediators
Conductive polymer/carbon scaffolds with clickable side chains	Coatings typically 100 nm–10 µm	Mechanically compliant; conductivity via percolation	eT is dominated by scaffold conductivity + ion transport	Can be improved by PEG/zwitterionic grafts	Use for textile/patch electrodes and high-strain zones.

**Table 3:** Critical comparison of orientation strategies for click-enabled bioreceptor immobilization, including their implications for accessibility, electron-transfer distance, and mechanical robustness [108,109,110].

Orientation Strategy	Bioreceptor Class and Required Engineering	Oriented Attachment Site and Handle Installed	Compatible Click Reaction (s)	Performance Rationale	Limitations and Validation Controls
Fc-binding affinity layer	Intact IgG (native); no direct chemical modification required	Orientation via Fc (or κ chain) capture. The click handle is typically installed on the affinity layer or electrode	CuAAC/SPAAC/IEDDA/Thiol-ene/yne photoclick	High fraction of antigen-binding sites accessible	Subclass/species dependence; noncovalent capture may desorb under sweat/shear
Fc glycan-directed coupling	Intact IgG; chemoenzymatic Fc N-glycan remodeling	Azide/alkyne installed on Fc glycan; attachment distal to Fab regions	SPAAC; CuAAC; IEDDA	Near-uniform orientation; minimal paratope perturbation	Glycan heterogeneity across species/subclasses
Engineered single cysteine	Recombinant IgG/Fab/scFv; site-directed mutagenesis or thiol re-bridging	Reactive thiol used to install azide/alkyne/DBCO/TCO via thiol-selective linkers	SPAAC/CuAAC/IEDDA; Thiol-ene/yne photoclick	Single-site coupling with tunable geometry	Avoid disulfide scrambling; control the degree of labeling
Sortase-mediated ligation	Recombinant proteins/antibodies bearing the LPXTG motif	Enzymatically append GGG-azide/alkyne/TCO at a defined terminus	CuAAC/SPAAC/IEDDA	Highly site-specific and modular; compatible with large proteins and antibodies	Requires tag engineering and enzyme handling; remove excess sortase/byproducts
Noncanonical amino acid (ncAA) incorporation	Recombinant proteins; requires engineered translation	Genetically encode azide/alkyne or strained-alkene handle at a single residue	CuAAC/SPAAC; IEDDA	True single-site handle with minimal post-processing	Expression yield/cost; potential folding effects, benchmark vs. wild-type
AviTag/BirA biotinylation + streptavidin capture	Recombinant proteins with AviTag at N/C terminus or exposed loop	Covalent biotin installed at AviTag. Oriented immobilization via (strept)avidin layer	Click optional: CuAAC/SPAAC/IEDDA to anchor (strept)avidin or biotin to the electrode	Highly specific and homogeneous; strong interaction supports mechanical robustness	(Strept)avidin adds distance; regeneration is difficult
DNA-directed immobilization (DDI)	Proteins/antibodies conjugated to ssDNA	ssDNA attached at a defined site; immobilization via hybridization provides controlled spacing/orientation	CuAAC/SPAAC/IEDDA	Chemically mild; programmable spacing	Salt-dependent; nuclease sensitivity
Tag-catcher covalent pairs	Recombinant fusion proteins; tag position sets orientation	Isopeptide-bond formation yields irreversible coupling. A catcher can be click-anchored to the electrode	CuAAC/SPAAC/IEDDA/Thiol-ene/yne photoclick	Exceptional mechanical stability; fast assembly;	Added protein bulk may increase tunneling distance; verify tag placement does not impair binding
Metal–affinity capture	Recombinant proteins/enzymes	NTA chelator assembled on electrode; His-tag binds with orientation defined by tag location	Click optional: anchor NTA–PEG-azide/alkyne via CuAAC/SPAAC/IEDDA	Reversible and mild; useful for screening/regeneration without harsh elution conditions	Lower stability than covalent routes
Aptamer end-tethering + mixed monolayer backfilling	DNA/RNA aptamers; synthetic end-modification (5′ or 3′)	Single-point attachment via terminal azide/alkyne/thiol; use diluents to tune density and avoid crowding	CuAAC/SPAAC; Thiol–Au SAM; Thiol-ene photoclick for polymeric substrates/patterning	Defined orientation; tunable distance improves target access and folding	Crowding reduces binding; backfilling affects SAM stability

Note: the click reactions listed refer to the covalent step that anchors the oriented bioreceptor (or an intermediate affinity scaffold) to the electrode. Abbreviations: Fc, fragment crystallizable; Fab, antigen-binding fragment; DBCO, dibenzocyclooctyne; BCN, bicyclononyne; TCO, trans-cyclooctene; MCH, 6-mercaptohexanol; OEG, oligo(ethylene glycol); NTA, nitrilotriacetic acid; DDI, DNA-directed immobilization.

**Table 4:** Decision framework for selecting handle installation and click-coupling routes across wearable and flexible substrate classes [142,143,144].

Substrate	Activation/Primer	Surface Handle (s) Installed	Click Reaction (s)	Wearable-Specific Guidance
Gold thin films on flexible carriers	Self-assembled monolayers (SAMs) of thiols; mixed SAMs with OEG/alkanol diluents	–N_3_, –C≡CH, strained alkyne, or tetrazine termini on alkanethiols	SPAAC or Tz–TCO IEDDA to CuAAC	Highest molecular-level control. Use mixed SAMs to avoid crowding; validate after bending/sweat exposure
Metal oxides and metal-coated foils	O_2_ plasma or UV/ozone hydroxylation followed by silanization	Azido-/alkynyl–silanes; Tz- or TCO–silanes for IEDDA; aminosilanes as a relay	SPAAC or IEDDA to CuAAC	Silanes are versatile but water-sensitive. Confirm monolayer formation and mechanical integrity. Prefer copper-free clicks for skin-contacting electrodes
Printed carbon and laser-induced graphene	Aryldiazonium electrografting; mild electro-oxidation to –COOH followed by amide coupling of propargylamine	Aryl–N_3_ or aryl–C≡CH motifs on carbon; alternatively, –COOH converted to alkyne-bearing amides	CuAAC to SPAAC	Control grafting density to avoid insulating multilayers. Diazonium layers can overgrow; use short electrografting times/pulse
Graphene/CNT films and papers	Controlled covalent functionalization or noncovalent π–π anchoring to preserve the sp^2^ lattice	Sparse –N_3_/alkyne handles on the lattice or on π–π adsorbed linkers (pyrene–alkyne, pyrene–N_3_)	SPAAC to CuAAC	Minimize defect density to preserve conductivity; quantify before/after functionalization. Use longer, flexible spacers to reduce strain transfer during bending
Conductive polymers on flexible electronics	Direct formation of a click-bearing polymer film, or deposit a clickable primer layer	Pendant –N_3_ (common) or alkyne groups integrated into polymer backbone/side chains	SPAAC to CuAAC	Enables conformal functionalization with minimal thickness penalty. Avoid harsh redox conditions that over-oxidize PEDOT
Silicone elastomers	O_2_ plasma to generate –Si–OH, then immediate silanization. Alternative: formulate with vinyl/norbornene groups for thiol-ene grafting	Surface –N_3_ or –C≡CH via silane monolayer; bulk/surface vinyl or norbornene groups for thiol-ene	SPAAC to Thiol-ene to CuAAC	Time-sensitive: PDMS hydrophobic recovery demands immediate silanization. For stretch, use compliant spacers and avoid brittle multilayers
Thermoplastic polymer films	O_2_ plasma/UV-ozone + adhesive primer	Commonly surface –N_3_ (via primer/polymer) or –C≡CH; for fastest wet coupling: Tz/TCO pairs in primer layer	SPAAC or IEDDA to Thiol-ene to CuAAC	Choose aqueous/solvent-minimized steps to prevent swelling/crazing. Consider antifouling co-functionalization
Textiles and cellulose-based substrates	Cellulose: C6-azidation or chemo-enzymatic oxidation + reductive amination to install –N_3_	Cellulose: surface –N_3_ or –C≡CH; synthetics: –N_3_/alkyne on coating/primer	SPAAC to CuAAC to Thiol-ene	Design for laundering/abrasion: favor covalent grafts + flexible spacers. If CuAAC is used, apply exhaustive rinsing + Cu chelation to minimize residual Cu
Hydrogel/bioadhesive interfaces	Build handles into the network (NB/ene + thiol; or Tz/TCO). Photopatterning or bulk gelation can introduce gradients/compartments	Norbornene/ene + thiols; tetrazine + TCO	Thiol-ene/yne photoclick to IEDDA	Prefer visible-light initiators for skin/biomolecule compatibility; avoid excessive radical load that can inactivate enzymes
MXenes and other emerging 2D materials	Exploit surface terminations for covalent derivatization; install azides via coupling chemistry	Surface –N_3_ or Tz/TCO, where compatible	SPAAC (metal-free, bioorthogonal coupling on nanosheets) to IEDDA	Protect from oxidation. Confirm functionalization does not collapse conductivity

## Data Availability

Data sharing does not apply to this article, as no new data were created or analyzed in this study.

## References

[1] Wu K.Y., Su M.E., Kim Y., Nguyen L., Marchand M., Tran S.D. (2025). Wearable biosensors: A comprehensive overview. Prog. Mol. Biol. Transl. Sci..

[2] Sletten E.M., Bertozzi C.R. (2009). Bioorthogonal chemistry: Fishing for selectivity in a sea of functionality. Angew. Chem. Int. Ed..

[3] Abdelfattah M.A., Jamali S.S., Kashaninejad N., Nguyen N.T. (2025). Wearable biosensors for health monitoring: Advances in graphene-based technologies. Nanoscale Horiz..

[4] Kalita N., Gogoi S., Minteer S.D., Goswami P. (2023). Advances in Bioelectrode Design for Developing Electrochemical Biosensors. ACS Meas. Sci. Au.

[5] Kim J., Campbell A.S., de Ávila B.E.F., Wang J. (2019). Wearable biosensors for healthcare monitoring. Nat. Biotechnol..

[6] Sassolas A., Blum L.J., Leca-Bouvier B.D. (2012). Immobilization strategies to develop enzymatic biosensors. Biotechnol. Adv..

[7] Cernat A., Tertiș M., Cristea C., Săndulescu R. (2015). Applications of Click Chemistry in the Development of Electrochemical Sensors. Int. J. Electrochem. Sci..

[8] Putzbach W., Ronkainen N.J. (2013). Immobilization Techniques in the Fabrication of Nanomaterial-Based Electrochemical Biosensors: A Review. Sensors.

[9] Mohamad Nor N., Ridhuan N.S., Abdul Razak K. (2022). Progress of Enzymatic and Non-Enzymatic Electrochemical Glucose Biosensor Based on Nanomaterial-Modified Electrode. Biosensors.

[10] Yüce M., Kurt H. (2017). How to make nanobiosensors: Surface modification and characterisation of nanomaterials for biosensing applications. RSC Adv..

[11] Sionkowska A., Kulka-Kamińska K., Brudzyńska P., Lewandowska K., Piwowarski Ł. (2024). The Influence of Various Crosslinking Conditions of EDC/NHS on the Properties of Fish Collagen Film. Mar. Drugs.

[12] Pawar M., Yadav P., H.N. G., Sai T.P., Gandhi S., Ghosh A. (2025). Challenges in Graphene-Based Biosensing: Exploring Critical Limitations and Strategies. ACS Sens..

[13] Beitello E., Osei K., Kobulnicky T., Breausche F., Friesen J.A., Driskell J.D. (2025). Oriented Surface Immobilization of Antibodies Using Enzyme-Mediated Site-Specific Biotinylation for Enhanced Antigen-Binding Capacity. Langmuir.

[14] Trilling A.K., Harmsen M.M., Ruigrok V.J.B., Zuilhof H., Beekwilder J. (2013). The effect of uniform capture molecule orientation on biosensor sensitivity: Dependence on analyte properties. Biosens. Bioelectron..

[15] Wang F., Xie Y., Zhu W., Wei T. (2023). Recent Advances in Functionalization Strategies for Biosensor Interfaces, Especially the Emerging Electro-Click: A Review. Chemosensors.

[16] Luu T., Gristwood K., Knight J.C., Jörg M. (2024). Click Chemistry: Reaction Rates and Their Suitability for Biomedical Applications. Bioconjugate Chem..

[17] Yang M., Wang S. (2025). Bioorthogonal Chemistry in Biomolecule Quantification: A Review of Reactions and Strategies. Chem.—A Eur. J..

[18] Tornøe C.W., Christensen C., Meldal M. (2002). Peptidotriazoles on Solid Phase: [1,2,3]-Triazoles by Regiospecific Copper(I)-Catalyzed 1,3-Dipolar Cycloadditions of Terminal Alkynes to Azides. J. Org. Chem..

[19] Kolanovic D., Pasupuleti R., Wallner J., Mlynek G., Wiltschi B. (2024). Site-Specific Immobilization Boosts the Performance of a Galectin-1 Biosensor. Bioconjugate Chem..

[20] Lu Z.Y., Chan Y.H. (2024). The importance of antibody orientation for enhancing sensitivity and selectivity in lateral flow immunoassays. Sens. Diagn..

[21] Hein J.E., Fokin V.V. (2010). Copper-catalyzed azide–alkyne cycloaddition (CuAAC) and beyond: New reactivity of copper(I) acetylides. Chem. Soc. Rev..

[22] Devaraj N.K., Decreau R.A., Ebina W., Collman J.P., Chidsey C.E.D. (2006). Rate of Interfacial Electron Transfer through the 1,2,3-Triazole Linkage. J. Phys. Chem. B.

[23] Fenoy G.E., Hasler R., Lorenz C., Movilli J., Marmisollé W.A., Azzaroni O., Huskens J., Bäuerle P., Knoll W. (2023). Interface Engineering of “Clickable” Organic Electrochemical Transistors toward Biosensing Devices. ACS Appl. Mater. Interfaces.

[24] Liu N., Song J., Lu Y., Davis J.J., Gao F., Luo X. (2019). Electrochemical Aptasensor for Ultralow Fouling Cancer Cell Quantification in Complex Biological Media Based on Designed Branched Peptides. Anal. Chem..

[25] Raza T., Qu L., Khokhar W.A., Andrews B., Ali A., Tian M. (2021). Progress of Wearable and Flexible Electrochemical Biosensors with the Aid of Conductive Nanomaterials. Front. Bioeng. Biotechnol..

[26] Han F., Wang T., Liu G., Liu H., Xie X., Wei Z., Li J., Jiang C., He Y., Xu F. (2022). Materials with Tunable Optical Properties for Wearable Epidermal Sensing in Health Monitoring. Adv. Mater..

[27] Jin Z., Yim W., Retout M., Housel E., Zhong W., Zhou J., Strano M.S., Jokerst J.V. (2024). Colorimetric sensing for translational applications: From colorants to mechanisms. Chem. Soc. Rev..

[28] Liu W., Cheng H., Wang X. (2023). Skin-interfaced colorimetric microfluidic devices for on-demand sweat analysis. Npj Flex. Electron..

[29] Yue X., Xu F., Zhang L., Ren G., Sheng H., Wang J., Wang K., Yu L., Wang J., Li G. (2022). Simple, Skin-Attachable, and Multifunctional Colorimetric Sweat Sensor. ACS Sens..

[30] Xiao J., Liu Y., Su L., Zhao D., Zhao L., Zhang X. (2019). Microfluidic Chip-Based Wearable Colorimetric Sensor for Simple and Facile Detection of Sweat Glucose. Anal. Chem..

[31] Zhang Y., Chu C.W., Ma W., Takahara A. (2020). Functionalization of Metal Surface via Thiol–Ene Click Chemistry: Synthesis, Adsorption Behavior, and Postfunctionalization of a Catechol- and Allyl-Containing Copolymer. ACS Omega.

[32] Liao A., Du W., Yang H. (2024). The Inverse Electron Demand Diels-Alder Reaction Between Tetrazine and Trans-Cyclooctene for Pretargeted Bioimaging Applications. Anal. Sens..

[33] Degirmenci A., Sanyal R., Sanyal A. (2024). Metal-Free Click-Chemistry: A Powerful Tool for Fabricating Hydrogels for Biomedical Applications. Bioconjug. Chem..

[34] Depienne S., Bouzelha M., Courtois E., Pavageau K., Lalys P.A., Marchand M., Alvarez-Dorta D., Nedellec S., Marín-Fernández L., Grandjean C. (2023). Click-electrochemistry for the rapid labeling of virus, bacteria and cell surfaces. Nat. Commun..

[35] Pineda-Castañeda H.M., Rivera-Monroy Z.J., Maldonado M. (2023). Copper(I)-Catalyzed Alkyne–Azide Cycloaddition (CuAAC) “Click” Reaction: A Powerful Tool for Functionalizing Polyhydroxylated Platforms. ACS Omega.

[36] Fenoy G.E., Hasler R., Quartinello F., Marmisollé W.A., Lorenz C., Azzaroni O., Bäuerle P., Knoll W. (2022). “Clickable” Organic Electrochemical Transistors. JACS Au.

[37] Chen M., Li Y., Han R., Chen Q., Jiang L., Luo X. (2022). Click reaction-assisted construction of antifouling immunosensors for electrochemical detection of cancer biomarkers in human serum. Sens. Actuators B Chem..

[38] Yáñez-Sedeño P., González-Cortés A., Campuzano S., Pingarrón J.M. (2019). Copper(I)-Catalyzed Click Chemistry as a Tool for the Functionalization of Nanomaterials and the Preparation of Electrochemical (Bio)Sensors. Sensors.

[39] Agouram N., El Hadrami E.M., Bentama A. (2021). 1,2,3-Triazoles as Biomimetics in Peptide Science. Molecules.

[40] Bonandi E., Christodoulou M.S., Fumagalli G., Perdicchia D., Rastelli G., Passarella D. (2017). The 1,2,3-triazole ring as a bioisostere in medicinal chemistry. Drug Discov. Today.

[41] Meldal M., Tomøe C.W. (2008). Cu-Catalyzed Azide−Alkyne Cycloaddition. Chem. Rev..

[42] Huang Y., Zhang P., Wang H., Chen Y., Liu T., Luo X. (2024). Genetic Code Expansion: Recent Developments and Emerging Applications. Chem. Rev..

[43] Fantoni N.Z., El-Sagheer A.H., Brown T. (2021). A Hitchhiker’s Guide to Click-Chemistry with Nucleic Acids. Chem. Rev..

[44] Song Y., Sun Q., Aguila B., Ma S. (2019). Opportunities of Covalent Organic Frameworks for Advanced Applications. Adv. Sci..

[45] Bu H.B., Götz G., Reinold E., Vogt A., Schmid S., Blanco R., Segura J.L., Bäuerle P. (2008). Click-functionalization of conducting poly(3,4-ethylenedioxythiophene) (PEDOT). Chem. Commun..

[46] Scavetta E., Mazzoni R., Mariani F., Margutta R.G., Bonfiglio A., Demelas M., Fiorilli S., Marzocchi M., Fraboni B. (2014). Dopamine amperometric detection at a ferrocene clicked PEDOT:PSS coated electrode. J. Mater. Chem. B.

[47] Kennedy D.C., McKay C.S., Legault M.C.B., Danielson D.C., Blake J.A., Pegoraro A.F., Stolow A., Mester Z., Pezacki J.P. (2011). Cellular Consequences of Copper Complexes Used To Catalyze Bioorthogonal Click Reactions. J. Am. Chem. Soc..

[48] Besanceney-Webler C., Jiang H., Zheng T., Feng L., Soriano Del Amo D., Wang W., Klivansky L.M., Marlow F.L., Liu Y., Wu P. (2011). Increasing the Efficacy of Bioorthogonal Click Reactions for Bioconjugation: A Comparative Study. Angew. Chem. Int. Ed. Engl..

[49] Guerrero S., Cadano D., Agüí L., Barderas R., Campuzano S., Yáñez-Sedeño P., Pingarrón J.M. (2019). Click chemistry-assisted antibodies immobilization for immunosensing of CXCL7 chemokine in serum. J. Electroanal. Chem..

[50] Uttamapinant C., Tangpeerachaikul A., Grecian S., Clarke S., Singh U., Slade P., Gee K.R., Ting A.Y. (2012). Fast, Cell-compatible Click Chemistry with Copper-chelating Azides for Biomolecular Labeling. Angew. Chem. Int. Ed. Engl..

[51] Devaraj N.K., Dinolfo P.H., Chidsey C.E.D., Collman J.P. (2006). Selective functionalization of independently addressed microelectrodes by electrochemical activation and deactivation of a coupling catalyst. J. Am. Chem. Soc..

[52] Sweedan A.O., Zhang K., Bashouti M.Y., Feichtner T. (2025). Large-Area Atomically Flat Monocrystalline Gold Flakes: Recent Advances, Applications, and Future Potential. arXiv.

[53] Park S., Bisht H., Park J., Park S., Hong Y., Chu D., Koh M., Lee H., Hong D. (2025). Linker-Engineered Tyrosine–Azide Coatings for Stable Strain-Promoted Azide–Alkyne Cycloaddition (SPAAC) Functionalization. Polymers.

[54] Baskin J.M., Prescher J.A., Laughlin S.T., Agard N.J., Chang P.V., Miller I.A., Lo A., Codelli J.A., Bertozzi C.R. (2007). Copper-free click chemistry for dynamic in vivo imaging. Proc. Natl. Acad. Sci. USA.

[55] Dommerholt J., Schmidt S., Temming R., Hendriks L.J.A., Rutjes F.P.J.T., Van Hest J.C.M., Lefeber D.J., Friedl P., van Delft F.L. (2010). Readily accessible bicyclononynes for bioorthogonal labeling and three-dimensional imaging of living cells. Angew. Chem. Int. Ed..

[56] Jewett J.C., Bertozzi C.R. (2010). Cu-free click cycloaddition reactions in chemical biology. Chem. Soc. Rev..

[57] Agard N.J., Prescher J.A., Bertozzi C.R. (2004). A strain-promoted [3 + 2] azide-alkyne cycloaddition for covalent modification of biomolecules in living systems. J. Am. Chem. Soc..

[58] Kim E., Koo H. (2019). Biomedical applications of copper-free click chemistry: In vitro, in vivo, and ex vivo. Chem. Sci..

[59] Dommerholt J., Rutjes F.P.J.T., Van Delft F.L., Vrabel M., Carell T., Nl F.V. (2016). Strain-Promoted 1,3-Dipolar Cycloaddition of Cycloalkynes and Organic Azides. Top. Curr. Chem..

[60] Wendeln C., Singh I., Rinnen S., Schulz C., Arlinghaus H.F., Burley G.A., Ravoo B.J. (2012). Orthogonal, metal-free surface modification by strain-promoted azide–alkyne and nitrile oxide–alkene/alkyne cycloadditions. Chem. Sci..

[61] Huang C.H., Hou S.Y., Severance S., Hwang C.C., Fang B.K., Gong M.M., Yu S.-L., Weng Y.-C., Wang L.-F., Dai C.-Y. (2023). Manipulating Diastereomeric Bicyclononynes to Sensitively Determine Enzyme Activity and Facilitate Macromolecule Conjugations. ACS Omega.

[62] Gautier C., López I., Breton T. (2021). A post-functionalization toolbox for diazonium (electro)-grafted surfaces: Review of the coupling methods. Mater. Adv..

[63] He Z., Wu J., Li W., Du Y., Lu L. (2024). Investigation of G-Quadruplex DNA-Mediated Charge Transport for Exploring DNA Oxidative Damage in Telomeres. Langmuir.

[64] Dommerholt J., Van Rooijen O., Borrmann A., Guerra C.F., Bickelhaupt F.M., Van Delft F.L. (2014). Highly accelerated inverse electron-demand cycloaddition of electron-deficient azides with aliphatic cyclooctynes. Nat. Commun..

[65] Mertgen A.S., Guex A.G., Tosatti S., Fortunato G., Rossi R.M., Rottmar M., Maniura-Weber K., Zürcher S. (2022). A low-fouling, self-assembled, graft co-polymer and covalent surface coating for controlled immobilization of biologically active moieties. Appl. Surf. Sci..

[66] Pringle T.A., Knight J.C. (2025). The effects of buffer, pH, and temperature upon SPAAC reaction rates. Org. Biomol. Chem..

[67] Collman J.P., Devaraj N.K., Eberspacher T.P.A., Chidsey C.E.D. (2006). Mixed Azide-Terminated Monolayers: A Platform for Modifying Electrode Surfaces. Langmuir.

[68] Knall A.C., Slugovc C. (2013). Inverse electron demand Diels-Alder (iEDDA)-initiated conjugation: A (high) potential click chemistry scheme. Chem. Soc. Rev..

[69] Darko A., Wallace S., Dmitrenko O., Machovina M.M., Mehl R.A., Chin J.W., Fox J.M. (2014). Conformationally Strained trans-Cyclooctene with Improved Stability and Excellent Reactivity in Tetrazine Ligation. Chem. Sci. (R. Soc. Chem. 2010).

[70] Handula M., Chen K.T., Seimbille Y. (2021). IEDDA: An Attractive Bioorthogonal Reaction for Biomedical Applications. Molecules.

[71] Blackman M.L., Royzen M., Fox J.M. (2008). The Tetrazine Ligation: Fast Bioconjugation based on Inverse-electron-demand Diels-Alder Reactivity. J. Am. Chem. Soc..

[72] Li Z., Cai H., Hassink M., Blackman M.L., Brown R.C.D., Conti P.S., Fox J.M. (2010). Tetrazine-trans-cyclooctene ligation for the rapid construction of 18F labeled probes. Chem. Commun..

[73] de Roode K.E., Rossin R., Robillard M.S. (2025). Toward Realization of Bioorthogonal Chemistry in the Clinic. Top. Curr. Chem..

[74] Yang M., Jalloh A.S., Wei W., Zhao J., Wu P., Chen P.R. (2014). Biocompatible click chemistry enabled compartment-specific pH measurement inside *E. coli*. Nat. Commun..

[75] Adhikari K., Vanermen M., Da Silva G., Van den Wyngaert T., Augustyns K., Elvas F. (2024). Trans-cyclooctene—A Swiss army knife for bioorthogonal chemistry: Exploring the synthesis, reactivity, and applications in biomedical breakthroughs. EJNMMI Radiopharm. Chem..

[76] Fang Y., Judkins J.C., Boyd S.J., am Ende C.W., Rohlfing K., Huang Z., Xie Y., Johnson D.S., Fox J.M. (2019). Studies on the Stability and Stabilization of trans-Cyclooctenes through Radical Inhibition and Silver (I) Metal Complexation. Tetrahedron.

[77] Liu L., Zhang D., Johnson M., Devaraj N.K. (2022). Light-activated tetrazines enable precision live-cell bioorthogonal chemistry. Nat. Chem..

[78] Eising S., Engwerda A.H.J., Riedijk X., Bickelhaupt F., Matthias, Bonger K. (2018). Article/Letter to editor. Bioconjug. Chem..

[79] Fang Y., Hillman A.S., Fox J.M. (2025). Advances in the Synthesis of Bioorthogonal Reagents: S-Tetrazines, 1,2,4-Triazines, Cyclooctynes, Heterocycloheptynes, and trans-Cyclooctenes. Bioorthogonal Reactions.

[80] Wang P., Na Z., Fu J., Tan C.Y.J., Zhang H., Yao S.Q., Sun H. (2014). Microarray immobilization of biomolecules using a fast trans-cyclooctene-tetrazine reaction. Chem. Commun..

[81] Patterson D.M., Nazarova L.A., Prescher J.A. (2014). Finding the Right (Bioorthogonal) Chemistry. ACS Chem. Biol..

[82] Chen Y., Kushner A.M., Williams G.A., Guan Z. (2012). Multiphase design of autonomic self-healing thermoplastic elastomers. Nat. Chem..

[83] Selvaraj R., Fox J.M. (2013). trans-Cyclooctene—A stable, voracious dienophile for bioorthogonal labeling. Curr. Opin. Chem. Biol..

[84] Alge D.L., Azagarsamy M.A., Donohue D.F., Anseth K.S. (2013). Synthetically Tractable Click Hydrogels for Three-Dimensional Cell Culture Formed Using Tetrazine–Norbornene Chemistry. Biomacromolecules.

[85] Yang Q., Hu Z., Rogers J.A. (2021). Functional Hydrogel Interface Materials for Advanced Bioelectronic Devices. Acc. Mater. Res..

[86] Oliveira B.L., Guo Z., Bernardes G.J.L. (2017). Inverse electron demand Diels–Alder reactions in chemical biology. Chem. Soc. Rev..

[87] Devaraj N.K., Weissleder R., Hilderbrand S.A. (2008). Tetrazine-Based Cycloadditions: Application to Pretargeted Live Cell Imaging. Bioconjugate Chem..

[88] McFarland J.M., Alečković M., Coricor G., Srinivasan S., Tso M., Lee J., Nguyen T.-H., Oneto J.M.M. (2023). Click Chemistry Selectively Activates an Auristatin Protodrug with either Intratumoral or Systemic Tumor-Targeting Agents. ACS Cent. Sci..

[89] Fischer M.J.E. (2010). Amine Coupling Through EDC/NHS: A Practical Approach. Methods Mol. Biol..

[90] Norberg O., Lee I.H., Aastrup T., Yan M., Ramström O. (2012). Photogenerated lectin sensors produced by thiol-ene/yne photo-click chemistry in aqueous solution. Biosens. Bioelectron..

[91] Krings N., Strehblow H.H., Kohnert J., Martin H.D. (2003). Investigations on the monolayer structure of thiol SAMs and the influence of conjugated π-bonds on the electronic molecular conductivity. Electrochim. Acta.

[92] Madhiri N. (2006). Proton Coupled Electron Transfer Kinetics of Redox Centers Proton Coupled Electron Transfer Kinetics of Redox Centers Attached to Self-Assembled Monolayers on Electrodes Attached to Self-Assembled Monolayers on Electrodes. Ph.D. Thesis.

[93] Bollella P., Katz E. (2020). Enzyme-based biosensors: Tackling electron transfer issues. Sensors.

[94] Hu Z., Zhu R., Figueroa-Miranda G., Zhou L., Feng L., Offenhäusser A., Mayer D. (2023). Truncated Electrochemical Aptasensor with Enhanced Antifouling Capability for Highly Sensitive Serotonin Detection. Biosensors.

[95] Frederix F., Bonroy K., Laureyn W., Reekmans G., Campitelli A., Dehaen W., Maes G. (2003). Enhanced Performance of an Affinity Biosensor Interface Based on Mixed Self-Assembled Monolayers of Thiols on Gold. Langmuir.

[96] Rapakousiou A., Deraedt C., Irigoyen J., Wang Y., Pinaud N., Salmon L., Ruiz J., Moya S., Astruc D. (2015). Synthesis and Redox Activity of “Clicked” Triazolylbiferrocenyl Polymers, Network Encapsulation of Gold and Silver Nanoparticles and Anion Sensing. Inorg. Chem..

[97] Weber K., Hockett L., Creager S. (1997). Long-Range Electronic Coupling between Ferrocene and Gold in Alkanethiolate-based Monolayers on Electrodes. J. Phys. Chem. B.

[98] Farjami E., Campos R., Ferapontova E.E. (2012). Effect of the DNA End of Tethering to Electrodes on Electron Transfer in Methylene Blue-Labeled DNA Duplexes. Langmuir.

[99] Chamkina E.S., Chamkin A.A., Knyazeva E.A., Ustynyuk N.A., Shifrina Z.B. (2024). Redox-Active Polyphenylene Dendrimers on the Way toward Efficient Electrochemical Sensors. ACS Appl. Polym. Mater..

[100] Acosta S., Chaskovska V., El-Maachi I., Englert J., Puertas-Bartolomé M., Jockenhoevel S., Rodríguez-Cabello J.C., Rodriguez-Emmenegger C., Fernández-Colino A. (2025). Bioorthogonal Mussel-Inspired Elastin-like Nanocoatings for Indwelling Devices. ACS Appl. Mater. Interfaces.

[101] Li J., Ye F., Wang W., Zhang D. (2025). Controlled synthesis of triazole polymers via ATRP/click chemistry reaction and preparation of their self-assembled film against copper corrosion. J. Coat. Technol. Res..

[102] Zhang H., Sun Z., Sun K., Liu Q., Chu W., Fu L., Dai D., Liang Z., Lin C.-T. (2025). Electrochemical Impedance Spectroscopy-Based Biosensors for Label-Free Detection of Pathogens. Biosensors.

[103] Trilling A.K., Hesselink T., Houwelingen Avan Cordewener J.H.G., Jongsma M.A., Schoffelen S., van Hest J.C., Zuilhof H., Beekwilder J. (2014). Orientation of llama antibodies strongly increases sensitivity of biosensors. Biosens. Bioelectron..

[104] Seo M.H., Han J., Jin Z., Lee D.W., Park H.S., Kim H.S. (2011). Controlled and Oriented Immobilization of Protein by Site-Specific Incorporation of Unnatural Amino Acid. Anal. Chem..

[105] Shen M., Rusling J.F., Dixit C.K. (2016). Site-Selective Orientated Immobilization of Antibodies and Conjugates for Immunodiagnostics Development. Methods.

[106] Prieto-Simón B., Saint C., Voelcker N.H. (2014). Electrochemical Biosensors Featuring Oriented Antibody Immobilization via Electrografted and Self-Assembled Hydrazide Chemistry. Anal. Chem..

[107] Kausaite-Minkstimiene A., Ramanaviciene A., Kirlyte J., Ramanavicius A. (2010). Comparative Study of Random and Oriented Antibody Immobilization Techniques on the Binding Capacity of Immunosensor. Anal. Chem..

[108] Yi W., Xiao P., Liu X., Zhao Z., Sun X., Wang J., Zhou L., Wang G., Cao H., Wang D. (2022). Recent advances in developing active targeting and multi-functional drug delivery systems via bioorthogonal chemistry. Signal Transduct. Target. Ther..

[109] Vilé G., Di Liberto G., Tosoni S., Sivo A., Ruta V., Nachtegaal M., Clark A.H., Agnoli S., Zou Y., Savateev A. (2022). Azide-Alkyne Click Chemistry over a Heterogeneous Copper-Based Single-Atom Catalyst. ACS Catal..

[110] Soler M., Lechuga L.M. (2021). Biochemistry strategies for label-free optical sensor biofunctionalization: Advances towards real applicability. Anal. Bioanal. Chem..

[111] Ricci F., Lai R.Y., Heeger A.J., Plaxco K.W., Sumner J.J. (2007). Effect of Molecular Crowding on the Response of an Electrochemical DNA Sensor. Langmuir.

[112] Love J.C., Estroff L.A., Kriebel J.K., Nuzzo R.G., Whitesides G.M. (2005). Self-Assembled Monolayers of Thiolates on Metals as a Form of Nanotechnology. Chem. Rev..

[113] Homaei A.A., Sariri R., Vianello F., Stevanato R. (2013). Enzyme immobilization: An update. J. Chem. Biol..

[114] Kim D., Herr A.E. (2013). Protein immobilization techniques for microfluidic assays. Biomicrofluidics.

[115] Chen H., Wang Z., Song Z.L., Fan G.C., Luo X. (2024). Click Conjugation of Zwitterionic Peptide with DNA Strand: An Efficient Antifouling Strategy for Versatile Photoelectrochemical Aptasensor. Anal. Chem..

[116] Ye H., Wang L., Huang R., Su R., Liu B., Qi W., He Z. (2015). Superior Antifouling Performance of a Zwitterionic Peptide Compared to an Amphiphilic, Non-Ionic Peptide. ACS Appl. Mater. Interfaces.

[117] Vasilescu A., Gáspár S., Gheorghiu M., Polonschii C., Banciu R.M., David S., Gheorghiu E., Marty J.-L. (2025). Promising Solutions to Address the Non-Specific Adsorption in Biosensors Based on Coupled Electrochemical-Surface Plasmon Resonance Detection. Chemosensors.

[118] Brothers M.C., Moore D., Lawrence M.S., Harris J., Joseph R.M., Ratcliff E., Ruiz O.N., Glavin N., Kim S.S. (2020). Impact of Self-Assembled Monolayer Design and Electrochemical Factors on Impedance-Based Biosensing. Sensors.

[119] Oberhaus F.V., Frense D., Beckmann D. (2020). Immobilization Techniques for Aptamers on Gold Electrodes for the Electrochemical Detection of Proteins: A Review. Biosensors.

[120] Blasi D., Sarcina L., Tricase A., Stefanachi A., Leonetti F., Alberga D., Mangiatordi G.F., Manoli K., Scamarcio G., Picca R.A. (2020). Enhancing the Sensitivity of Biotinylated Surfaces by Tailoring the Design of the Mixed Self-Assembled Monolayer Synthesis. ACS Omega.

[121] Roy D., Park J.W. (2015). Spatially nanoscale-controlled functional surfaces toward efficient bioactive platforms. J. Mater. Chem. B.

[122] Lee H., Reginald S.S., Sravan J.S., Lee M., Chang I.S. (2025). Advanced strategies for enzyme–electrode interfacing in bioelectrocatalytic systems. Trends Biotechnol..

[123] Nitzan A. (2001). Electron transmission through molecules and molecular interfaces. Annu. Rev. Phys. Chem..

[124] Codelli J.A., Baskin J.M., Agard N.J., Bertozzi C.R. (2008). Second-Generation Difluorinated Cyclooctynes for Copper-Free Click Chemistry. J. Am. Chem. Soc..

[125] Zheng T., Rouhanifard S.H., Jalloh A.S., Wu P. (2012). Click Triazoles for Bioconjugation. Top. Heterocycl. Chem..

[126] Wang J., Zhou Q., Howard C.B., Trau M. (2025). Physicochemical Tuning of Bispecific Antibody Fragment Immobilization for Robust Digital SERS Biosensing. Anal. Chem..

[127] Bednar R.M., Golbek T.W., Kean K.M., Brown W.J., Jana S., Baio J.E., Karplus P.A., Mehl R.A. (2019). Immobilization of Proteins with Controlled Load and Orientation. ACS Appl. Mater. Interfaces.

[128] Khasbaatar A., Xu Z., Lee J.H., Campillo-Alvarado G., Hwang C., Onusaitis B.N., Diao Y. (2023). From Solution to Thin Film: Molecular Assembly of π-Conjugated Systems and Impact on (Opto)electronic Properties. Chem. Rev..

[129] Meier M.F.T., Thetiot F., Pittala N., Lieberwirth I., Kunzler C., Triki S., Jonas U. (2021). Thermoresponsive polymers as macromolecular coordination ligands: Complexation-dependence of thermally induced aggregation in aqueous solution. Polym. Chem..

[130] Minagawa Y., Nakata S., Date M., Ii Y., Noji H. (2022). On-Chip Enrichment System for Digital Bioassay Based on Aqueous Two-Phase System. ACS Nano.

[131] Yang B., Wang Y., Vorobii M., Sauter E., Koenig M., Kumar R., Rodriguez-Emmenegger C., Hirtz M. (2022). Evaluation of Dibenzocyclooctyne and Bicyclononyne Click Reaction on Azido-Functionalized Antifouling Polymer Brushes via Microspotting. Adv. Mater. Interfaces.

[132] Nicosia C., Huskens J. (2013). Reactive self-assembled monolayers: From surface functionalization to gradient formation. Mater. Horiz..

[133] Simonian A., Arugula M.A., Bollella P. (2025). Immobilization and Surface Chemistry. Biosensors as Analytical Tools for the 21st Century.

[134] Xing T., Wang X., Xu Y., Sun F., Chen M., Yan Q., Ma Z., Jiang H., Chen X., Li X. (2024). Click method preserves but EDC method compromises the therapeutic activities of the peptide-activated hydrogels for critical ischemic vessel regeneration. Biomed. Pharmacother..

[135] Liu Y., Canoura J., Alkhamis O., Xiao Y. (2021). Immobilization strategies for enhancing sensitivity of electrochemical aptamer-based sensors. ACS Appl. Mater. Interfaces.

[136] Sánchez-Tirado E., González-Cortés A., Yáñez-Sedeño P., Pingarrón J.M. (2016). Carbon nanotubes functionalized by click chemistry as scaffolds for the preparation of electrochemical immunosensors. Application to the determination of TGF-beta 1 cytokine. Analyst.

[137] Dutta N., Lillehoj P.B., Estrela P., Dutta G. (2021). Electrochemical Biosensors for Cytokine Profiling: Recent Advancements and Possibilities in the Near Future. Biosensors.

[138] Qi H., Ling C., Huang R., Qiu X., Shangguan L., Gao Q., Zhang C. (2012). Functionalization of single-walled carbon nanotubes with protein by click chemistry as sensing platform for sensitized electrochemical immunoassay. Electrochim. Acta.

[139] Song J., Luo Y., Hao Z., Qu M., Huang C., Wang Z., Yang J., Liang Q., Jia Y., Song Q. (2025). Graphene-based wearable biosensors for point-of-care diagnostics: From surface functionalization to biomarker detection. Mater. Today Bio.

[140] Jin Z., McNicholas T.P., Shih C.J., Wang Q.H., Paulus G.L.C., Hilmer A.J., Shimizu S., Strano M.S. (2011). Click Chemistry on Solution-Dispersed Graphene and Monolayer CVD Graphene. Chem. Mater..

[141] Mathew M., Radhakrishnan S., Vaidyanathan A., Chakraborty B., Rout C.S. (2020). Flexible and wearable electrochemical biosensors based on two-dimensional materials: Recent developments. Anal. Bioanal. Chem..

[142] Park S., Park K., Kim T.Y., Jo Y., Kim J., Son K., Choi H., Ok M., Shin S.R., Seo J. (2026). Mechanically Robust and Anti-Biofouling Hybrid Encapsulation via Layered Organic–Liquid Interfaces for Implantable Devices. Small.

[143] Choi Y.J., Lee S., Kim Y., Park S., Kim H.J. (2025). High-Performance Electrochemical Adhesives Enabled by Perfluorinated Sulfonic-Acid Ionomers with Precise Adhesion Control and Long-Term Switchability. Adv. Sci..

[144] Lee S., Akerson A., Pardakhtim R., Hajiesmaili E., Rhodes K., Li Z., Stanley A., Amini A., Piazza D., Daraio C. (2025). Deformation Driven Suction Cups: A Mechanics-Based Approach to Wearable Electronics. Adv. Sci..

[145] Li W., Li Y., Xu K. (2019). Azidated Graphene: Direct Azidation from Monolayers, Click Chemistry, and Bulk Production from Graphite. Nano Lett..

[146] Nxele S.R., Mashazi P., Nyokong T. (2015). Electrode Modification Using Alkynyl Substituted Fe(II) Phthalocyanine via Electrografting and Click Chemistry for Electrocatalysis. Electroanalysis.

[147] Wepasnick K.A., Smith B.A., Schrote K.E., Wilson H.K., Diegelmann S.R., Howard Fairbrother D. (2011). Surface and structural characterization of multi-walled carbon nanotubes following different oxidative treatments. Carbon.

[148] Ferrier D.C., Honeychurch K.C. (2021). Carbon Nanotube (CNT)-Based Biosensors. Biosensors.

[149] CPaulus G.L., Hua Wang Q., Strano M.S. (2013). Covalent Electron Transfer Chemistry of Graphene with Diazonium Salts. Acc. Chem. Res..

[150] Xin M., Li J., Ma Z., Pan L., Shi Y. (2020). MXenes and Their Applications in Wearable Sensors. Front. Chem..

[151] Ali M., Hasan E., Barman S.C., Hedhili M.N., Alshareef H.N., Alsulaiman D. (2024). Peptide nucleic acid-clicked Ti_3_C_2_T_x_ MXene for ultrasensitive enzyme-free electrochemical detection of microRNA biomarkers. Mater. Horiz..

[152] Hadhri M., Colleoni C., D’Agostino A., Erhaim M., Rosa R.P., Rosace G., Trovato V. (2025). Eco-Friendly Octylsilane-Modified Amino-Functional Silicone Coatings for a Durable Hybrid Organic–Inorganic Water-Repellent Textile Finish. Polymers.

[153] Gökaltun A., Kang Y.B., Yarmush M.L., Usta O.B., Asatekin A. (2019). Simple Surface Modification of Poly(dimethylsiloxane) via Surface Segregating Smart Polymers for Biomicrofluidics. Sci. Rep..

[154] Liu Y., He T., Gao C. (2005). Surface modification of poly(ethylene terephthalate) via hydrolysis and layer-by-layer assembly of chitosan and chondroitin sulfate to construct cytocompatible layer for human endothelial cells. Colloids Surf. B Biointerfaces.

[155] Hillborg H., Gedde U.W. (1998). Hydrophobicity recovery of polydimethylsiloxane after exposure to corona discharges. Polymer.

[156] Arima Y., Iwata H. (2007). Effect of wettability and surface functional groups on protein adsorption and cell adhesion using well-defined mixed self-assembled monolayers. Biomaterials.

[157] Beal J.H.L., Bubendorfer A., Kemmitt T., Hoek I., Mike Arnold W. (2012). A rapid, inexpensive surface treatment for enhanced functionality of polydimethylsiloxane microfluidic channels. Biomicrofluidics.

[158] Nongbe M.C., Bretel G., Ekou L., Ekou T., Robitzer M., Le Grognec E., Felpin F.-X. (2018). Cellulose paper azide as a molecular platform for versatile click ligations: Application to the preparation of hydrophobic paper surface. Cellulose.

[159] Xu C., Spadiut O., Araújo A.C., Nakhai A., Brumer H. (2012). Chemo-enzymatic assembly of clickable cellulose surfaces via multivalent polysaccharides. ChemSusChem.

[160] Degirmenci A., Sanyal R., Sanyal A. (2024). ‘Clickable’ polymeric coatings: From antibacterial surfaces to interfaces with cellular and biomolecular affinity. RSC Applied Polymers.

[161] Fairbanks B.D., Macdougall L.J., Mavila S., Sinha J., Kirkpatrick B.E., Anseth K.S., Bowman C.N. (2021). Photoclick Chemistry: A Bright Idea. Chem. Rev..

[162] Szymańska A., Przybylak M., Przybylska A., Maciejewski H. (2025). Silanization of Cotton Fabric to Obtain Durable Hydrophobic and Oleophobic Materials. Int. J. Mol. Sci..

[163] Derikvand F., Yin D.L.T., Barrett R., Brumer H. (2016). Cellulose-Based Biosensors for Esterase Detection. Anal. Chem..

[164] Li H., Papadakis R. (2020). Click Chemistry Enabling Covalent and Non-Covalent Modifications of Graphene with (Poly)saccharides. Polymers.

[165] Li M., Liu M., Qi F., Lin F.R., Jen A.K.Y. (2024). Self-Assembled Monolayers for Interfacial Engineering in Solution-Processed Thin-Film Electronic Devices: Design, Fabrication, and Applications. Chem. Rev..

[166] Awasthi S., De S., Pandey S.K. (2024). Surface Grafting of Carbon Nanostructures. Handbook of Functionalized Carbon Nanostructures: From Synthesis Methods to Applications.

[167] Kotoulas K.T., Campbell J., Skirtach A.G., Volodkin D., Vikulina A. (2022). Surface Modification with Particles Coated or Made of Polymer Multilayers. Pharmaceutics.

[168] Mueller E., Poulin I., Bodnaryk W.J., Hoare T. (2022). Click Chemistry Hydrogels for Extrusion Bioprinting: Progress, Challenges, and Opportunities. Biomacromolecules.

[169] Liu L., Ni Y., Mao J., Li S., Ng K.H., Chen Z., Huang J., Cai W., Lai Y. (2022). Flexible and Highly Conductive Textiles Induced by Click Chemistry for Sensitive Motion and Humidity Monitoring. ACS Appl. Mater. Interfaces.

[170] Aleksic M., Meng X. (2024). Protein Haptenation and Its Role in Allergy. Chem. Res. Toxicol..

[171] Ochtrop P., Hackenberger C.P.R. (2020). Recent advances of thiol-selective bioconjugation reactions. Curr. Opin. Chem. Biol..

[172] Payne M.E., Kareem O.O., Williams-Pavlantos K., Wesdemiotis C., Grayson S.M. (2021). Mass spectrometry investigation into the oxidative degradation of poly(ethylene glycol). Polym. Degrad. Stab..

[173] Bandodkar A.J., Gutruf P., Choi J., Lee K.H., Sekine Y., Reeder J.T., Jeang W.J., Aranyosi A.J., Lee S.P., Model J.B. (2019). Battery-free, skin-interfaced microfluidic/electronic systems for simultaneous electrochemical, colorimetric, and volumetric analysis of sweat. Sci. Adv..

[174] Koh A., Kang D., Xue Y., Lee S., Pielak R.M., Kim J., Hwang T., Min S., Banks A., Bastien P. (2016). A soft, wearable microfluidic device for the capture, storage, and colorimetric sensing of sweat. Sci. Transl. Med..

[175] Gao W., Emaminejad S., Nyein H.Y.Y., Challa S., Chen K., Peck A., Fahad H.M., Ota H., Shiraki H., Kiriya D. (2016). Fully integrated wearable sensor arrays for multiplexed in situ perspiration analysis. Nature.

[176] Bilbao E., Garate O., Rodríguez Campos T., Roberti M., Mass M., Lozano A., Longinotti G., Monsalve L., Ybarra G. (2023). Electrochemical Sweat Sensors. Chemosensors.

[177] Venrooij K.R., de Bondt L., Bonger K.M. (2024). Mutually Orthogonal Bioorthogonal Reactions: Selective Chemistries for Labeling Multiple Biomolecules Simultaneously. Top Curr. Chem..

[178] Zou S., Peng G., Ma Z. (2024). Surface-Functionalizing Strategies for Multiplexed Molecular Biosensing: Developments Powered by Advancements in Nanotechnologies. Nanomaterials.

